# Mental health of LGBTQ+ workers: a systematic review

**DOI:** 10.1186/s12888-025-06556-2

**Published:** 2025-02-11

**Authors:** Dunya Tomic, Monica O’Dwyer, Tessa Keegel, Karen Walker-Bone

**Affiliations:** Monash Centre for Occupational and Environmental Health, School of Public Health and Preventive Medicine, Monash University, Melbourne, Australia

**Keywords:** Mental health, Depression, Anxiety, LGBTQ+, Lesbian, Gay, Bisexual, Transgender, Workplace, Systematic review

## Abstract

**Background:**

Members of the lesbian, gay, bisexual, transgender, queer, and other sexual and gender minorities (LGBTQ+) community have greater risk of mental health disorders compared to the general population, however most evidence is from young people. We sought to systematically review and summarise the evidence for the burden and risk of mental health disorders.

**Methods:**

A PRISMA-compliant literature search was conducted in MEDLINE, Embase, PsycInfo, Scopus, and CINAHL for articles published from 2000 to 2024. Quantitative or mixed-methods studies that reported mental health outcomes among LGBTQ+ workers were included. Effect measures included prevalence and odds ratios, with variations in mental health across occupations and specific sexual or gender minorities reported where possible. This systematic review was prospectively registered through PROSPERO (CRD42024587605).

**Results:**

Out of 5736 unique articles screened, 33 articles (32 individual studies) fulfilled eligibility criteria, including 8369 LGBTQ+ workers. Other than studies of sex workers, only 12 studies had research questions specific to LGBTQ+ workers’ mental health. Most studies (30/32) were cross-sectional and reported increased odds of depression, anxiety, or suicidality among LGBTQ+ compared to non-LGBTQ+ workers. Prevalence estimates and methodology varied widely. Factors associated with adverse mental health outcomes among select groups of LGBTQ+ workers included internalised stigma, heterosexism, job stress and low income. We found no studies comparing workers across industries and no studies involving workplace interventions.

**Conclusions:**

There are limited objective data regarding LGBTQ+ workers’ mental health. Given the heterogeneity of the LGBTQ+ population, dedicated longitudinal research into the mental health of specific sexual and gender minorities across all industries and occupations is needed to determine causal factors, the impact of intersectionality, and the effectiveness of workplace interventions.

**Supplementary Information:**

The online version contains supplementary material available at 10.1186/s12888-025-06556-2.

## Background

Members of the lesbian, gay, bisexual, transgender, queer, and other sexual and gender minorities (LGBTQ+) community face discrimination and victimisation in many areas of everyday life, including in public spaces, such as schools and workplaces, and when accessing health services [1]. They are often subject to social stress, social exclusion, stigma including within their own families, homophobic or transphobic hatred and violence, and internalised shame and stigma about their sexual or gender identity [2]. All these experiences contribute to negative mental health consequences, including an increased burden of depression, anxiety, substance use disorders, suicidality and suicide attempts among LGBTQ+ populations compared to heterosexual counterparts [3–5]. The LGBTQ+ community is heterogeneous and specific groups, such as transgender people, experience specific and more intense forms of discrimination and harassment [6]. Most research of LGBTQ+ mental health has been conducted among adolescents and youth, with less data on working-aged adults [7–9].

Safe and fulfilling work is associated with physical and mental health benefits [10]. However, given the substantial portion of daily life occupied by work among most adults, when stigma is experienced in the workplace it may be especially pervasive, inescapable, and damaging to mental health [11]. As early as 1999, researchers have argued that the workplace is an important environment to study the minority stress experienced by LGBTQ+ individuals [12]. Studies of LGBTQ+ workers, mostly drawing on qualitative evidence, have identified an increased burden of adverse workplace experiences such as hiring discrimination, harassment, work stress, and decreased job satisfaction, compared to the general workforce [13–15]. However, there is still a paucity of data regarding the impact of these experiences on LGBTQ+ workers’ mental health, and no published review to date has specifically described the evidence on this topic. It is essential for businesses and public health decision-makers to understand the burden and risk of mental health disorders among LGBTQ+ workers in order for relevant interventions to be designed and implemented. As such, we sought to systematically review and summarise studies published in the academic literature since 2000 describing LGBTQ+ workers’ mental health, including variation between occupations and subgroups of the LGBTQ+ population.

## Methods

### Search strategy

In the conceptualisation stage of the review, we consulted with individuals who identified as LGBTQ+ workers through relevant research and mental health advocacy networks. We asked them to review the draft scope and aims of the study and the terminology to ensure that it was appropriate and sensitive. A search strategy was devised, with input from a librarian with search strategy expertise. The search was conducted on September 9, 2024, and was limited to studies published in English from January 1, 2000. Five electronic databases were searched: Ovid MEDLINE, Embase, PsycInfo, Scopus, and CINAHL.

The databases were searched with three sets of keywords combined with the AND operator. The first set included LGBTQ+ terms (lesbian, gay, bisexual, transgender, sexual and gender minorities, sexual orientation). The second set included variations of workplace terms (workplace, work, occupation, employee). The third set of terms related to mental health (mental health, psychological health), plus any specific International Classification of Diseases, Tenth Revision (ICD-10) conditions that have been reported in relevant reviews of LGBTQ+ mental health in the last 10 years [9, 16–18]. All terms were used as keywords as well as being matched to subject headings. Terms relating to non-binary, asexual and intersex identities were captured under broader LGBTQ+ MeSH terms included in the search. For example, “gender nonbinary”, “intersex”, “intersex persons”, and “asexuality” were all captured under MeSH searches in the databases investigated.

The full search strategies for all databases including MeSH search field qualifiers can be found in the Supplementary Appendix. Retrieved results were imported into Endnote, de-duplicated, and then uploaded into Covidence, a web-based tool that streamlines the screening process for systematic reviews.

### Inclusion and exclusion criteria

Eligibility criteria were devised using the PICO/PECO framework (population = LGBTQ+, intervention/exposure = work; comparison = non-LGBTQ+; outcome = mental health) [19, 20]. Inclusion criteria were: study population included and reported specifically on LGBTQ+ workers (or workers from any sexual or gender minority group); and mental health outcomes including depression, anxiety, substance use disorders, post-traumatic stress disorder (PTSD), eating disorders, body dysmorphia, bipolar disorder, personality disorders, schizophrenia, and suicide (attempted or completed, including suicidal ideation or plans). Workers were defined as any individual currently receiving income for services, in any employment arrangement including sex workers and gig economy workers. Quantitative or mixed-methods cross-sectional studies, cohort studies, case-control studies, and randomised controlled trials were included.

Exclusion criteria were studies of the general workforce with no specific results for any sexual or gender minority workers; studies of LGBTQ+ community members with no specification of working status; studies of school or university students (or studies of workplaces that included students where no separate analysis was conducted for only workers); studies of military personnel (which do not use standard occupational terms and some of which would not be captured without including terms such as ‘military’, which would introduce bias into the search strategy without using terms for all specific occupations; these studies have otherwise already been summarised in existing narrative reviews of LGBTQ+ military personnel [21, 22]); and measures of workplace wellbeing that do not constitute ICD-10 mental health conditions (e.g., burnout, job stress, job strain, harassment, bullying, job dissatisfaction, psychological distress, substance use without any indication of substance use disorder). Study types that were excluded were qualitative, commentaries, editorials, opinion pieces, conference proceedings or abstracts, university theses or dissertations, theoretical frameworks, case reports, and systematic or narrative reviews. The bibliographies of all systematic and narrative reviews were hand-searched for any additional original studies that met the inclusion criteria and had not been already identified for screening.

### Review protocol

This review was performed in accordance with the updated Preferred Reporting Items for Systematic Reviews and Meta-Analyses (PRISMA) guidelines (Supplementary Table 1) [23] and prospectively registered in PROSPERO (CRD42024587605). All retrieved articles were screened independently at the title and abstract level by two reviewers per article (authors DT, MOD, and TK). All full-text articles identified as being potentially relevant in title and abstract screening were then screened by two reviewers independently. Any discrepancies at the title and abstract or full-text stage were resolved through discussion or adjudication by a third reviewer. A data extraction form was devised, and data were extracted into a Microsoft Excel spreadsheet by one author (DT) with a second author (TK or MOD) checking all extraction. The categories for extraction were author; year; country; criminalisation of same-sex activity (yes/no) [24]; study type; participant details; mental health outcomes; methods for measuring outcomes; and results. Effect measures included prevalence, odds ratios (OR), relative risks (RR), and prevalence ratios (PR), adjusted for confounders and with 95% confidence intervals where applicable.

### Risk of bias assessment

A risk of bias assessment was conducted using Johanna Briggs Institute criteria [25] for all included studies. Specific criteria were used for each study type. An overall rating of low, moderate, or high risk of bias for each study was determined based on the assessment of critical domains. The determination of whether a domain was critical depended on the context of the study and the study design. For example, some studies with overall rating of high risk of bias may have only had one high risk rating in a critical domain, whilst others may have had multiple unclear ratings in several domains. Each article was independently assessed by two reviewers (authors DT and TK), with any discrepancies resolved through a consensus process. Inclusion of articles in the systematic review was not based on the results of the risk of bias assessments.

## Results

**Fig. 1:**
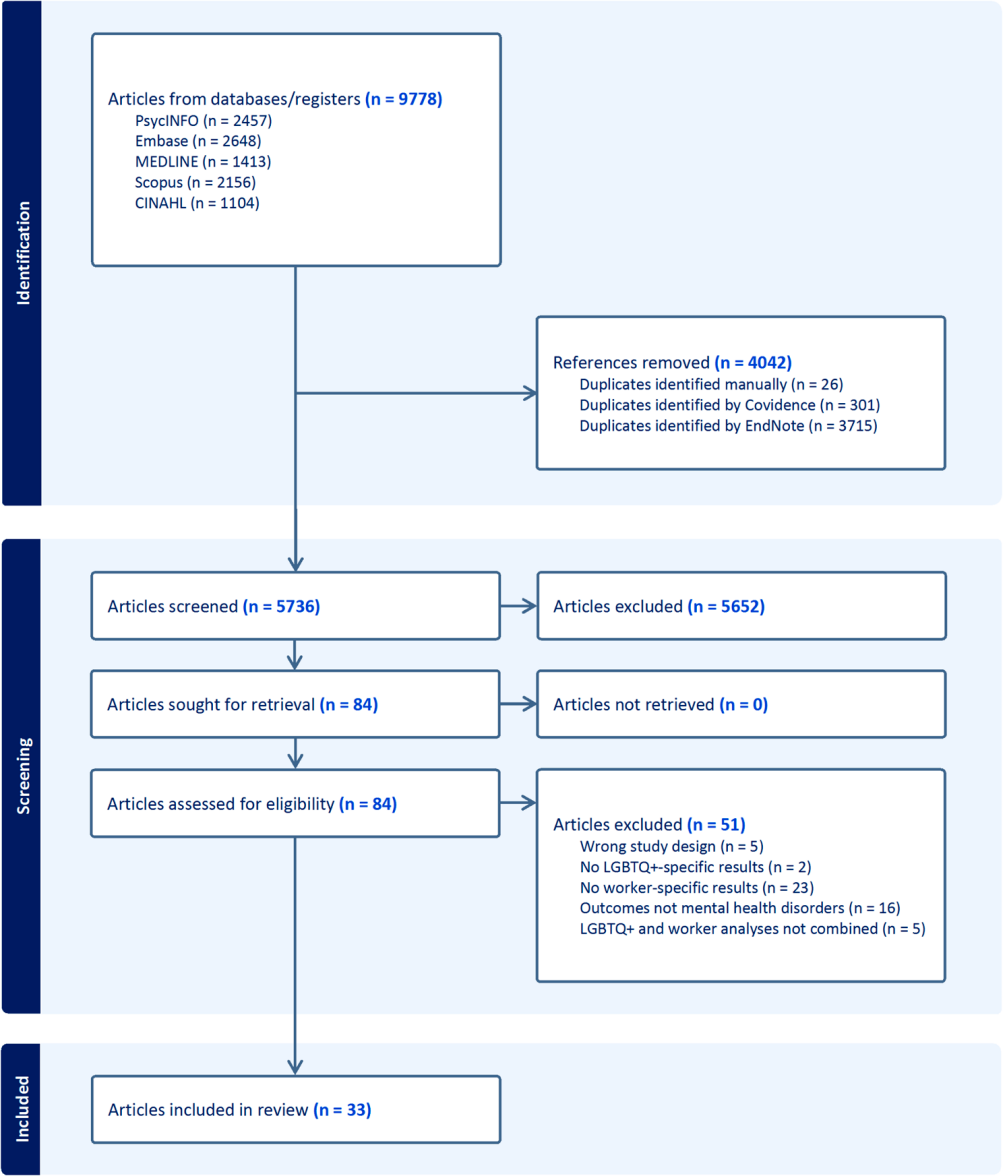
Preferred reporting items for systematic reviews and meta-analyses (PRISMA) flow diagram

After removal of all duplicates, 5736 articles were screened. A total of 84 articles deemed potentially eligible for inclusion were screened through to full text level, of which 51 articles were excluded (Supplementary Table 2). Therefore, 33 articles were identified for inclusion (Fig. 1), which corresponded to 32 individual studies (Table 1) with a total of 8369 LGBTQ+ workers. None of the reviews that were screened contained any additional references that met the inclusion criteria for this review. Almost all included studies (30/32) were cross-sectional, with few (2/32) cohort studies. There were 14 studies from the United States, three from China, two each from Brazil, India and the United Kingdom, and one each from Pakistan, the Czech Republic, the Dominican Republic, Australia, Jamaica, Spain, Canada, Malaysia, and Thailand. Three studies came from countries where same-sex activity is still criminalised (Jamaica, Malaysia, Pakistan). The most common cohort of workers studied was sex workers (13/32). Other specific occupations explored in more than one study were healthcare workers (5/32), emergency services workers (2/32), and frontline workers during the COVID-19 pandemic (2/32). There was one study of each of farmers, schoolteachers, and veterinary professionals, while seven studies were of the general workforce with occupations not specified.

**Table 1:** Description of the 32 included studies reporting mental health outcomes in LGBTQ+ workers

Author, year	Country	Criminalisation of same-sex activity [24]?	Study type	Specifically studying LGBTQ+ workers?	Sample size, *n*(LGBTQ+ workers)/ *n*(total cohort)	Characteristics of LGBTQ+ workers	Outcomes
Ali, 2023 [41]	Pakistan	Yes	Cross-sectional	Yes	91/91	MSM and transgender community health workers	Depression
Amsalem, 2023 [45]	United States	No	Cohort study	No	51/2545	Transgender essential workers during COVID-19 pandemic (including healthcare, manufacturing, food industry, construction, transportation, hospitality, emergency services)	Anxiety, depression
Bar-Johnson, 2014 [47]	Czech Republic	No	Cross-sectional	Yes	23/40	Homosexual and bisexual male sex workers	Anxiety, depression
Brogan, 2003 [28]	United States	No	Cross-sectional	Yes	115/4292	Lesbian physicians	Alcohol abuse/ dependence, depression, eating disorders, substance abuse/ dependence
Chandler, 2021 [34]	United States	No	Cross-sectional	Yes	245/4421	Black MSM sex workers	Depression
Chang, 2019 [42]	China	No	Cross-sectional	Yes	198/198	Transgender women sex workers	Depression
Cuthbertson, 2024 [29]	United States	No	Cross-sectional	Yes	148/148	LGBTQ+ farmers	Anxiety, depression
Day, 2024 [54]	United States	No	Cross-sectional	Yes	422/840	LGB workers aged 25 years and above	PTSD
de Mattos Russo Rafael, 2021 [53]	Brazil	No	Cross-sectional	No	167/345	Transgender women sex workers	Suicidal ideation and attempts
Goldberg, 2013 [43]	United States	No	Cross-sectional	Yes	172/172	Lesbian and gay dual-earner couples with adopted children	Anxiety, depression
Goldenberg, 2021 [27]	Dominican Republic	No	Cross-sectional	Yes	100/100	Transgender women sex workers living with HIV	Anxiety, depression
Griffin, 2023 [89]	United States	No	Cross-sectional	No	858/1090	Full-time or part-time employed LGBTQ+ individuals	Depression
Klare, 2021 [30]	United States	No	Cross-sectional	Yes	167/1188	LGBTQ+ employed adults	Anxiety, depression
Kyron, 2021 [48]	Australia	No	Cross-sectional	Yes	1087/14,536	LGBQ+ emergency services employees	Suicidal plans, thoughts, and attempts
Lee, 2019 [56]	United Kingdom	No	Cross-sectional	Yes	105/105	LGBT schoolteachers	Seeking help for depression or anxiety, time off work for depression or anxiety
Logie, 2017 [35]	Jamaica	Yes	Cross-sectional	Yes	42/137	Transgender women sex workers	Depression
Luz, 2024 [50]	Brazil	No	Cross-sectional	No	274/4148	Bisexual and homosexual physicians	Suicidal plans and attempts
Moya, 2020 [31]	Spain	No	Cross-sectional	Yes	229/366	LGBTI workers	Depression
Nuttbrock, 2014 [36]	United States	No	Cohort study	No	NS/230	Transgender women sex workers aged 19–59 years	Depression
Puri, 2017 [55]	Canada	No	Cross-sectional	No	177/692	SGM sex workers	Any mental health disorder (depression, PTSD, anxiety, schizophrenia, borderline personality disorder, attention-deficit/ hyperactivity disorder, bipolar disorder, other diagnosis specified)
Rashid, 2023 [90]	Malaysia	Yes	Cross-sectional	Yes	141/141	Full-time or part-time employed transgender women	Anxiety, depression
Renkiewicz, 2022 [52]	United States	No	Cross-sectional	No	60/681	Sexual minority emergency medical services personnel	Suicidality
Scoresby, 2023 [49]	United States	No	Cross-sectional	No	478/2208	LGBTQ+ veterinary professionals	Suicidal thoughts, plans, and attempts
She, 2022 [26]	China	No	Cross-sectional	Yes	199/199	Transgender women sex workers who were identified as being at risk of mental health issues	Anxiety, depression, suicidal ideation
Smith, 2004 [44]	United States	No	Cross-sectional	Yes	97/97	LGB workers	Depression
Srivastava, 2022 & 2023 [37, 38]	India	No	Cross-sectional	Yes	1897/3548	Transgender women and MSM sex workers	Depression
Sugg, 2021 [32]	United States	No	Cross-sectional	No	81/3045	Non-binary and transgender frontline or essential workers during COVID-19 pandemic who engaged with a text-based crisis service	Depression, substance abuse, suicidal thoughts
Teoh, 2023 [51]	United Kingdom	No	Cross-sectional	No	65/424	LGB junior doctors	Suicidal ideation
Thirunavukkarasu, 2021 [39]	India	No	Cross-sectional	No	108/235	MSM sex workers	Anxiety, depression
Wojcik, 2022 [33]	United States	No	Cross-sectional	Yes	102/741	SGM frontline healthcare workers	Alcohol use disorder, anxiety, depression, PTSD
Yan, 2014 [40]	China	No	Cross-sectional	Yes	200/404	MSM sex workers	Depression
Yasami, 2023 [46]	Thailand	No	Cross-sectional	Yes	270/270	Gay men and transgender women sex workers	Anxiety, depression

HIV = human immunodeficiency virus; LGB = lesbian, gay, and bisexual; LGBT = lesbian, gay, bisexual, and transgender; LGBTI = lesbian, gay, bisexual, transgender, and intersex; LGBTQ+ = lesbian, gay, bisexual, transgender, queer, and other sexual and gender minorities; LGBQ+ = lesbian, gay, bisexual, and queer; MSM = men who have sex with men; NS = not stated; PTSD = post-traumatic stress disorder; SGM = sexual and gender minority

**Fig. 2:**
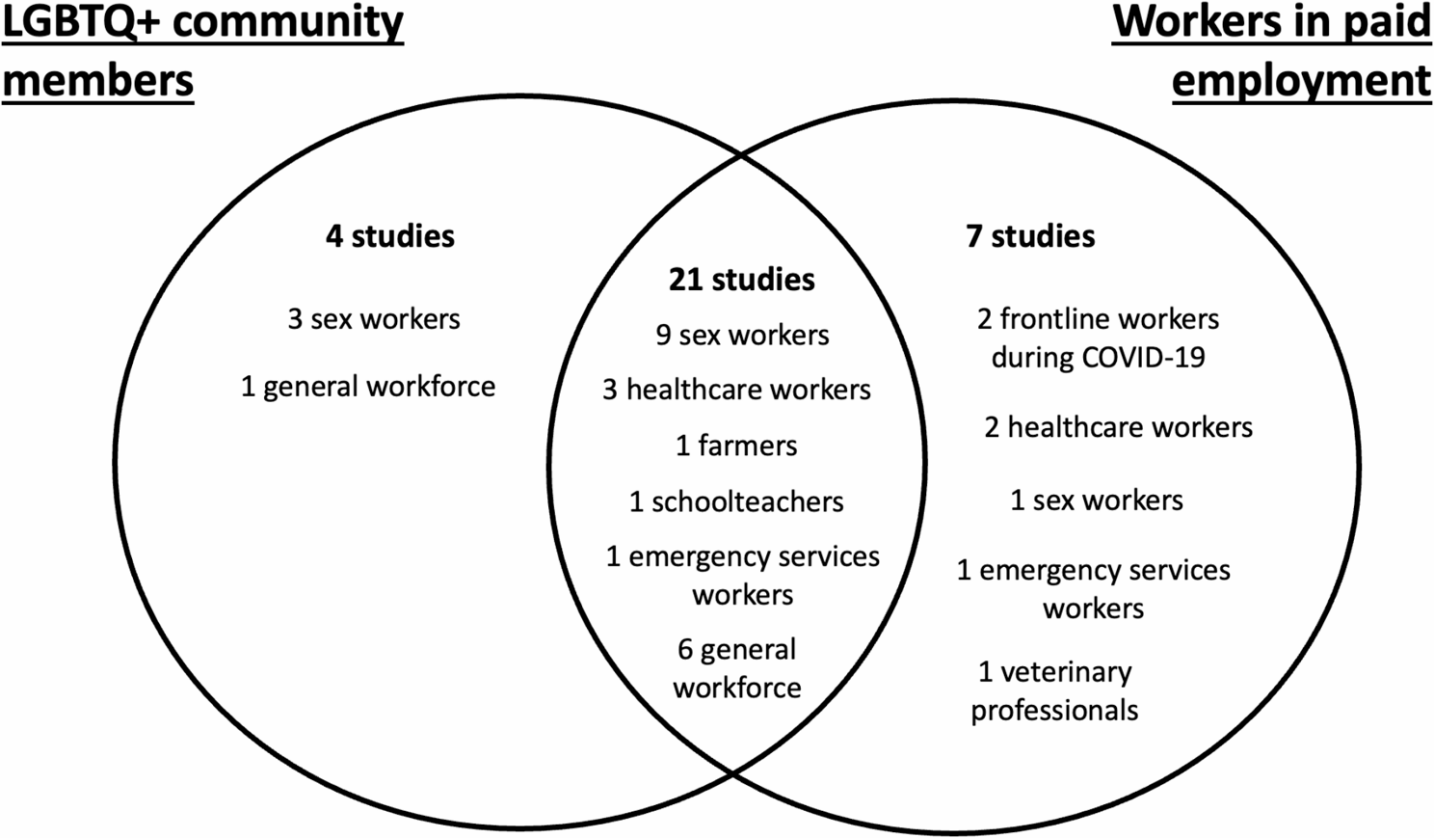
Overlap between specific focus on LGBTQ+ and working populations among included studies

Other than studies of sex workers (13 studies), the research question was only specific to mental health of LGBTQ+ workers in 12 studies. The remainder either looked at mental health of the general LGBTQ+ community and reported some results specifically for workers, or looked at the mental health of the general workforce and reported some results specifically for LGBTQ+ individuals (Fig. 2). The largest population of LGBTQ+ workers was 1897 and the smallest was 23. The most common mental health outcome was depression, reported in 23 studies. Other outcomes reported by more than one study were: anxiety (11/32), suicidality or suicide attempts (8/32), alcohol and/or other substance abuse (3/32), and PTSD (2/32). One study explored each of: eating disorders, a composite outcome including any mental health disorder, and outcomes of access to help or time off work for either depression or anxiety.

### Depression

Overall estimated prevalence rates for depression among LGBTQ+ workers ranged from 24 to 86.7%. However, sample bias and differences in population and screening instruments undermine any meaningful comparison between groups. Work-related associations of depression in select groups of LGBTQ+ workers included workplace heterosexism, high job stress, and low supervisor support. Low income and divorced or widowed marital status were also associated with depression in transgender women sex workers.

Of the 23 studies reporting on depression, 16 reported prevalence estimates for LGBTQ+ workers (Table 2). Eight different methods (seven screening tools and self-report depression) were used, with different cut-off scores used for some screening tools. The highest estimate of 89.7% was reported in a sample who had already scored above a pre-defined cut-off for depression, anxiety, or suicidal ideation and was therefore a highly selected and biased sample [26]. The lowest depression prevalence of 24% was reported among 100 transgender women sex workers living with human immunodeficiency virus (HIV) in the Dominican Republic [27]. Additionally, 12 studies reported significantly higher depression prevalence and/or severity either among LGBTQ+ workers compared to non-LGBTQ+ [28–33] or sex workers compared to other LGBTQ+ individuals [34–40].

**Table 2:** Results of the included studies reporting on depression among LGBTQ+ workers

Author, year	Characteristics of LGBTQ+ workers	Methods for measuring depression	Pertinent findings
Ali, 2023 [41]	91 MSM and transgender community health workers	PHQ-9 ≥ 10	Prevalence: 31.8%. Associations: Previous psychiatric illness (*p* < 0.001), history of deliberate self-harm (*p* = 0.022), history of suicide attempts (*p* = 0.015), increasing SJSS scores (*p* = 0.011), decreasing GSE scores (*p* = 0.005).
Amsalem, 2023 [45]	51 transgender essential workers during COVID-19 pandemic	PHQ-9	Other: Mean PHQ-9 scores consistently highest among transgender respondents (range 10.5–11.1) and lowest in cisgender men (range 6.5–6.7).
Bar-Johnson, 2014 [47]	Nine homosexual and 14 bisexual male sex workers	BDI > 10	Prevalence: 33% in homosexuals, 42% in bisexuals, 47% in heterosexuals.
Brogan, 2003 [28]	115 lesbian physicians	Self-report	Prevalence: 33.3% in lesbians, 19.4% in heterosexuals. Comparison: *p* = 0.01.
Chandler, 2021 [34]	245 Black MSM	CESD-10 ≥ 10	Prevalence: 59.4% in sex workers, 22.2% in other MSM. Comparison: aOR 3.88 (95% CI 2.91–5.19).
Chang, 2019 [42]	198 transgender women sex workers	PHQ-9 ≥ 10	Prevalence: 25.3%. Associations: Marital status (OR 2.36 for divorced or widowed vs. unmarried, 95% CI 1.08–5.15), monthly income (OR 0.34 for >$900 vs. <$450, 95% CI 0.14–0.84), HIV status (OR 0.39 for negative vs. positive. 95% CI 0.19–0.78).
Cuthbertson, 2024 [29]	148 LGBTQ+ farmers	PHQ-8 ≥ 10, self-report	Prevalence: 50.7% self-report, 36.1% PHQ-8. Comparison: Higher prevalence of PHQ-8 depression in transgender vs. non-transgender (*p* = 0.043), gay vs. non-gay (*p* = 0.045), queer vs. non-queer (*p* < 0.001). Other: 25.8% with depression on PHQ-8 had not been diagnosed.
Goldberg, 2013 [43]	47 lesbian and 39 gay dual-earner couples with adopted children	CESD	Associations: Low levels of supervisor support (*p* = 0.012).
Goldenberg, 2021 [27]	100 transgender women sex workers living with HIV	PHQ-9 ≥ 10	Prevalence: 24%. Associations: Internalised sex work stigma (aOR 1.19, 95% CI 1.03–1.36), social cohesion (aOR 0.81, 95% CI 0.71–0.95).
Griffin, 2023 [89]	215 full-time and 643 part-time employed LGBTQ+ individuals	PHQ-9 ≥ 15	Prevalence: 39.3% in full-time, 35.0% in part-time.
Klare, 2021 [30]	167 LGBTQ+ employed adults	PHQ-9 ≥ 10	Prevalence: 39.5% in LGBTQ+, 19.0% in heterosexual. Comparison: *p* < 0.001.
Logie, 2017 [35]	42 transgender women sex workers	PHQ-2 ≥ 3	Comparison: aOR 1.7 for sex workers vs. transgender women not in sex work (95% CI 1.1–2.6)
Moya, 2020 [31]	229 LGBTI workers	CESD-7	Comparison: Mean CESD-7 1.90 in LGBTI, 1.86 in heterosexual (*p* < 0.001). Other: Relationship between sexual orientation and depression entirely accounted for by job discrimination and work stress (*p* = 0.50 in mediation models).
Nuttbrock, 2014 [36]	Transgender women sex workers aged 19–59 years (number not stated)	MINI	Comparison: aOR 2.50 for sex workers vs. transgender women not in sex work (95% CI 1.58–3.94). Other: Smaller effect size when psychological and physical gender abuse included as covariates (aOR 2.16, 95% CI 1.20–3.91).
Rashid, 2023 [90]	141 full-time or part-time employed transgender women	DASS-21 (cut-off unclear)	Prevalence: 33.3%.
She, 2022 [26]	199 transgender women sex workers at risk of mental health problems (subsample 1, *n* = 126: scored above cut-off for probable depression or anxiety or had suicidal ideation; subsample 2, *n* = 109: perceived needs for mental health services; overlap between participants)	CESD ≥ 16	Prevalence: 89.7% in subsample 1, 70.6% in subsample 2.
Smith, 2004 [44]	97 LGB workers	CESD ≥ 16	Associations: Heterosexism (*p* < 0.01). Positive relationship between blaming responses and depression at low levels of heterosexism (*p* < 0.05) but not at high levels.
Srivastava, 2022 [37]	952 transgender women and 945 MSM sex workers	CESD-10 ≥ 10	Comparison: aOR 1.41 for transgender women sex workers vs. transgender women not in sex work (95% CI 1.07–1.86), aOR 1.43 for MSM sex workers vs. MSM not in sex work (95% CI 1.18–1.75).
Srivastava, 2023 [38]	952 transgender women sex workers	CESD-10 ≥ 10	Prevalence: 42.1% in transgender women sex workers, 28.9% in transgender women not in sex work. Comparison: aOR 1.40 for transgender women sex workers vs. transgender women not in sex work (95% CI 1.05–1.84).
Sugg, 2021 [32]	20 non-binary and 61 transgender frontline or essential workers during COVID-19 pandemic who engaged with a text-based crisis service	Daily text conversations flagged for depression	Comparison: Non-binary vs. male aOR 2.54 (95% CI 1.04–6.24), transgender vs. male aOR 1.05 (95% CI 0.61–1.82).
Thirunavukkarasu, 2021 [39]	108 MSM sex workers	CESD ≥ 16	Prevalence: 51.9% in sex workers, 39.4% in other MSM. Comparison: *p* = 0.055.
Wojcik, 2022 [33]	102 SGM frontline healthcare workers	PHQ-9 ≥ 10	Prevalence: 35.9% in SGM, 20.7% in non-SGM. Comparison: aOR 1.887 for SGM vs. non-SGM (95% CI 1.127–3.161).
Yan, 2014 [40]	200 MSM sex workers	CESD-12 ≥ 9	Prevalence: 70.0% in sex workers, 46.1% in other MSM. Comparison: aOR 1.86 for sex workers vs. other MSM (95% CI 1.07–3.24).
Yasami, 2023 [46]	270 gay men and transgender women sex workers	DASS-21 ≥ 10	Prevalence: 86.7%.

aOR = adjusted odds ratio; BDI = Beck’s Depression Inventory; CESD = Centre for Epidemiological Studies Depression Scale; CI = confidence intervals; DASS-21 = 21-item Depression, Anxiety, and Stress Scale; GSE = General Self Efficacy questionnaire; HIV = human immunodeficiency virus; LGB = lesbian, gay, and bisexual; LGBT = lesbian, gay, bisexual, and transgender; LGBTI = lesbian, gay, bisexual, transgender, and intersex; LGBTQ+ = lesbian, gay, bisexual, transgender, queer, and other sexual and gender minorities; MINI = Mini International Neuropsychiatric Interview; MSM = men who have sex with men; OR = odds ratio; PHQ = Patient Health Questionnaire; SGM = sexual and gender minority; SJSS = Subjective Job Stress Scale

Five studies reported factors associated with depression among LGBTQ+ workers, all in small cohorts of less than 200 workers [27, 41–43]. Work-related factors associated with depression included: increasing subjective job stress scale (SJSS) scores among 91 men who have sex with men (MSM) and transgender community health workers (*p* = 0.011) [41]; low levels of supervisor support among 47 lesbian and 39 gay dual-earner couples (*p* = 0.012) [43]; and workplace heterosexism in a general sample of 97 lesbian, gay, and bisexual (LGB) workers (*p* < 0.01) [44]. Additional associations of depression were described in a study of 198 transgender women sex workers, which compared divorced or widowed marital status to unmarried (OR 2.36, 95% CI 1.08–5.15) and monthly income >$900 to <$450 (OR 0.34, 95% CI 0.14–0.84) [42]. A further four studies reported other findings relating to depression [29, 31, 36, 45]. This included the finding that 25.8% of 148 LGBTQ+ farmers who were flagged as having probable depression on the Patient Health Questionnaire (PHQ-8) had not self-reported a history of depression [29].

### Anxiety

Estimated prevalence rates for anxiety among LGBTQ+ workers varied greatly from 0 to 80%. As for depression, heterogeneity in study population characteristics and methods used to define anxiety precluded making any generalisable estimates for the broader LGBTQ+ workforce. Only two studies explored risk factors for anxiety, which included low supervisor support in the general LGBTQ+ workforce and internalised stigma among sex workers.

Nine of the 11 studies reporting on anxiety presented prevalence estimates for generalised anxiety disorder (GAD), with widely variable methods (Table 3). The highest prevalence of 80% came from a study of 270 sex workers (gay men and transgender women) [46] while the lowest prevalence of 0% was reported in nine homosexual male sex workers [47]. One study reported social anxiety disorder prevalence of 26.2% and 24.8% in two cohorts of transgender women sex workers, however this was the biased sample (described above) in which the cohort was selected on the basis of high depression or anxiety scores [26]. Five studies compared prevalence of GAD in LGBTQ+ workers to non-LGBTQ+ [29, 30, 33, 43, 47]. Some pertinent findings included significantly more anxiety symptoms in bisexual compared to both homosexual (*p* = 0.002) and heterosexual (*p* = 0.012) male sex workers [47], lower anxiety prevalence in non-binary compared to binary farmers (*p* = 0.008) [29], and higher levels of anxiety in gay compared to lesbian dual-earner couples with adopted children (*p* = 0.036) [43].

**Table 3:** Results of the included studies reporting on anxiety among LGBTQ+ workers

Author, year	Characteristics of LGBTQ+ workers	Methods for measuring anxiety	Pertinent findings
Amsalem, 2023 [45]	51 transgender essential workers during COVID-19 pandemic	GAD-7	Other: Mean GAD-7 scores consistently highest among transgender respondents (range 9.2–10.5) followed by cisgender women (range 7.4–8.1) and lowest among cisgender men (range 5.3–5.7).
Bar-Johnson, 2014 [47]	Nine homosexual and 14 bisexual male sex workers	Zung’s Self-Rating Anxiety Scale > 45	Prevalence: 0% in homosexuals, 28% in bisexuals, 17% in heterosexuals. Comparison: Bisexuals significantly higher in anxiety symptoms than homosexuals (*p* = 0.002) and heterosexuals (*p* = 0.012).
Cuthbertson, 2024 [29]	148 LGBTQ+ farmers	GAD-7 ≥ 8, self-report	Prevalence: 56.9% self-report, 46.2% GAD-7. Comparison: Lower prevalence in non-binary compared to binary (*p* = 0.008), queer compared to non-queer (*p* < 0.001). Higher prevalence in transgender compared to non-transgender (*p* = 0.03). Other: 26.6% with anxiety on GAD-7 had not been diagnosed.
Goldberg, 2013 [43]	47 lesbian and 39 gay dual-earner couples with adopted children	20-item state anxiety subscale of STAI	Comparison: Higher levels of anxiety in gay compared to lesbian (*p* = 0.036). Associations: Low levels of supervisor support (*p* = 0.028).
Goldenberg, 2021 [27]	100 transgender women sex workers living with HIV	HADS-A > 7	Prevalence: 34%. Associations: Internalised sex work stigma (aOR 1.12, 95% CI 1.02–1.23), social cohesion (aOR 0.86, 95% CI 0.75–0.98).
Klare, 2021 [30]	167 LGBTQ+ employed adults	GAD-7 ≥ 10	Prevalence: 11.4% in LGBTQ+, 4.2% in heterosexual. Comparison: *p* < 0.001.
Rashid, 2023 [90]	141 full-time or part-time employed transgender women	DASS-21 (cut-off unclear)	Prevalence: 48.2%.
She, 2022 [26]	199 transgender women sex workers at risk of mental health problems (subsample 1, *n* = 126: scored above cut-off for probable depression or anxiety or had suicidal ideation; subsample 2, *n* = 109: perceived needs for mental health services; overlap between participants)	GAD-7 ≥ 5 for GAD Mini-SPIN ≥ 6 for social anxiety disorder	Prevalence: GAD 79.4% in subsample 1, 68.8% in subsample 2. Social anxiety disorder 26.2% in subsample 1, 24.8% in subsample 2.
Thirunavukkarasu, 2021 [39]	108 MSM sex workers	GAD-7 ≥ 10	Prevalence: 49.1% in sex workers, 28.3% in other MSM. Comparison: *p* = 0.055.
Wojcik, 2022 [33]	102 SGM frontline healthcare workers	GAD-7 ≥ 7	Prevalence: 48.9% in SGM, 32.9% in non-SGM. Comparison: aOR 1.807 for SGM vs. non-SGM (95% CI 1.096–2.976).
Yasami, 2023 [46]	270 gay men and transgender women sex workers	DASS-21 ≥ 10	Prevalence: 80%.

DASS-21 = 21-item Depression, Anxiety, and Stress Scale; GAD = Generalised Anxiety Disorder; GAD-7 = Generalised Anxiety Disorder 7-item Scale; HADS-A = Hospital Anxiety and Depression Scale Anxiety Module; HIV = human immunodeficiency virus; LGBTQ+ = lesbian, gay, bisexual, transgender, queer, and other sexual and gender minorities; Mini-SPIN = Mini-Social Phobia Inventory; MSM = men who have sex with men; PHQ = Patient Health Questionnaire; SGM = sexual and gender minority; STAI = State-Trait Anxiety Inventory

Only two studies explored factors associated with anxiety among LGBTQ+ workers, with positive associations reported for low levels of workplace supervisor support (*p* = 0.028) and internalised sex work stigma (aOR 1.12, 95% CI 1.02–1.23), while better social cohesion was associated with less anxiety (aOR 0.86, 95% CI 0.75–0.98) [27, 43]. Other associations described with anxiety included consistently highest mean GAD 7-item scale (GAD-7) scores among 79 transgender essential workers during the COVID-19 pandemic (range 9.2–10.5) compared to cisgender women (range 7.4–8.1) and cisgender men (range 5.3–5.7) [45], and 26.6% of 148 LGBTQ+ farmers with GAD-7 anxiety not having been previously diagnosed [29].

### Suicidality and suicide attempts

Suicidality was reported in 0-64.8% of workers from specific sexual or gender minorities and/or occupational groups, while the prevalence of suicide attempts ranged from 0 to 12.4%. Workers who identified as genderfluid, agender, bisexual, asexual, or who were questioning their sexual orientation, reported some of the highest rates of suicidality, while high-risk occupations included emergency services personnel, especially fire and rescue personnel.

A total of eight studies reported on suicidality or suicide attempts (Table 4). The most comprehensive data came from two cross-sectional studies from Australia and the United States, both of which used self-report measures of prevalence. The Australian study included 1087 lesbian, gay, bisexual, and queer (LGBQ+) emergency services employees (ambulance, fire and rescue, and police) and found that all measures of suicidality were consistently higher among LGBQ+ workers compared to heterosexual workers [48]. Fire and rescue LGBQ+ workers were generally the worst affected; 12.4% had attempted suicide and were almost six times as likely to have done so compared to heterosexual workers (aOR 5.93, 95% CI 2.7–13.2). The United States study of 478 LGBTQ+ veterinary professionals also explored suicidal thoughts, plans, and attempts, reporting prevalence for a wide range of specific sexual and gender minority groups [49]. Some of the key findings included the highest prevalence of suicidal thoughts among genderfluid (52.9%) and agender (42.9%) workers, and a significantly higher likelihood of suicidal thoughts (*p* < 0.001) and plans (*p* = 0.033) among bisexual, fluid, queer, questioning, asexual, or other sexual orientation compared to monosexual workers. A study of physicians also found a significant association between bisexuality and suicidal plans or attempts (aOR 5.083 vs. heterosexual, 95% CI 2.54–10.16) [50]. Two further studies found increased odds of suicidality among LGBTQ+ workers compared to heterosexual workers [51, 52] while a study of non-binary or transgender frontline workers found no association [32]. The final two studies were of sex workers: one found no increase in suicidal ideation among 167 transgender women sex workers compared to other transgender women [53] while the other reported prevalence of suicidal ideation of 23.9-27% in the biased sample who already scored above probable depression, anxiety, or suicidal ideation thresholds [26].

**Table 4:** Results of the included studies reporting on suicidality and suicide attempts among LGBTQ+ workers

Author, year	Characteristics of LGBTQ+ workers	Outcomes	Methods for measuring outcomes	Pertinent findings
de Mattos Russo Rafael, 2021 [53]	167 transgender women sex workers	Suicidal ideation and attempts	Self-report	Comparison: Suicidal ideation- aPR 0.76 for sex workers vs. other transgender women (95% CI 0.61–0.95). Suicide attempts- PR (unadjusted) 0.90 (95% CI 0.63–1.27).
Kyron, 2021 [48]	1087 LGBQ+ emergency services employees	Suicidal thoughts, plans, and attempts	Self-report	Prevalence: Suicidal thoughts (ever)- Ambulance 33.6%, Fire and rescue 30.6%, Police 20.6%, Total 26.4%. Suicidal thoughts (past 12mo)- Ambulance 13.3%, Fire and rescue 15.4%, Police 11.0%, Total 12.6%. Suicidal plans (ever)- Ambulance 18.3%, Fire and rescue 19.8%, Police 9.9%, Total 14.6%. Suicidal plans (past 12mo)- Ambulance 6.8%, Fire and rescue 8.7%, Police 3.9%, Total 6.4%. Suicide attempts (ever)- Ambulance 7.1%, Fire and rescue 12.4%, Police 5.1%, Total 7.1%. Comparison: LGBQ+ vs. heterosexual. Suicidal thoughts (ever)- Ambulance aOR 2.41 (95% CI 1.8–3.2), Fire and rescue aOR 2.75 (1.7–4.5), Police aOR 1.70 (1.3–2.3), Total aOR 2.06 (95% CI 1.7–2.5). Suicidal thoughts (past 12mo)- Ambulance aOR 2.01 (95% CI 1.2–3.0), Fire and rescue aOR 2.11 (95% CI 1.1–4.0), Police aOR 1.73 (95% CI 1.2–2.5), Total aOR 1.88 (95% CI 1.5–2.4). Suicidal plans (ever)- Ambulance aOR 2.44 (95% CI 1.7–3.5), Fire and rescue aOR 3.46 (95% CI 1.9–6.5), Police aOR 1.60 (95% CI 1.1–2.4), Total aOR 2.10 (95% CI 1.6–2.7). Suicidal plans (past 12mo)- Ambulance aOR 2.03 (95% CI 1.2–3.5), Fire and rescue aOR 2.43 (95% CI 1.1–5.3), Police aOR 1.51 (95% CI 0.8–2.9), total aOR 1.90 (95% CI 1.3–2.7). Suicide attempts (ever)- Ambulance aOR 2.46 (95% CI 1.4–4.8), Fire and rescue aOR 5.93 (95% CI 2.7–13.2), police aOR 2.04 (95% CI 1.2–2.7), Total aOR 2.69 (95% CI 1.3–4.1).
Luz, 2024 [50]	59 bisexual and 215 homosexual physicians	Suicidal plans and attempts (composite outcome)	Self-report	Prevalence: 32.2% in bisexuals, 13.0% in homosexuals, 8.1% in heterosexuals. Comparison: Bisexual vs. heterosexual aOR 5.083 (95% CI 2.544–10.158), homosexual vs. heterosexual aOR 1.402 (95% CI 0.886–2.219).
Renkiewicz, 2022 [52]	60 sexual minority emergency medical services personnel	Suicidality	Self-report	Prevalence: 34.9% in homosexuals, 22.9% in heterosexuals, 64.8% in those with “other” sexual orientation. Comparison: Sexual minority vs. heterosexual aOR 2.22 (95% CI 1.16–4.25).
Scoresby, 2023 [49]	478 LGBTQ+ veterinary professionals	Suicidal thoughts, plans, and attempts	Self-report	Prevalence: Thoughts- (by gender) cisgender female 16.4%, cisgender male 11.9%, transgender female 21.9%, transgender male 36.4%, gender nonconforming 33.3%, genderfluid 52.9%, agender 42.9%, questioning 33.3%, other 19.1% (by sexual orientation) heterosexual 13.8%, homosexual 16.3%, bisexual/non-monosexual 36.4%, fluid 38.5%, queer 21.9%, questioning 29.6%, other 36.4%, asexual 23.5%. Plans- (by gender) cisgender female 4.1%, cisgender male 4.6%, transgender female 1.4%, transgender male 0.0%, gender nonconforming 6.1%, genderfluid 5.9%, agender 14.3%, questioning 8.3%, other 4.3% (by sexual orientation) heterosexual 3.3%, homosexual 2.3%, bisexual/non-monosexual 9.5%, fluid 0.0%, queer 3.1%, questioning 0.0%, other 9.1%, asexual 5.9%. Attempts- (by gender) cisgender female 0.9%, cisgender male 0.9%, transgender female 0%, transgender male 0%, gender nonconforming 0%, genderfluid 0%, agender 0%, questioning 0%, other 0% (by sexual orientation) heterosexual 0.7%, homosexual 0%, bisexual/ non-monosexual 1.5%, fluid 0%, queer 0%, questioning 0%, other 0%, asexual 2.9%. Comparison: Bisexual, fluid, queer, questioning, asexual, or other more likely to have suicidal thoughts (*p* < 0.001) and to make a suicide plan (*p* = 0.033).
She, 2022 [26]	199 transgender women sex workers at risk of mental health problems (subsample 1, *n* = 126: scored above cut-off for probable depression or anxiety or had suicidal ideation; subsample 2, *n* = 109: perceived needs for mental health services; overlap between participants)	Suicidal ideation	Self-report	Prevalence: 27.0% in subsample 1, 23.9% in subsample 2.
Sugg, 2021 [32]	20 non-binary and 61 transgender frontline or essential workers during COVID-19 pandemic who engaged with a text-based crisis service	Suicidal thoughts	Daily text conversations flagged for suicidal thoughts	Comparison: Non-binary vs. male aOR 1.35 (95% CI 0.52–3.51), transgender vs. male aOR 1.19 (95% CI 0.65–2.20).
Teoh, 2023 [51]	65 LGB junior doctors	Suicidal ideation	Paykel Suicidality Scale, yes to any of final three items	Comparison: LGB vs. non-LGB aOR 2.18 (95% CI 1.14–4.12).

aOR = adjusted odds ratio; aPR = adjusted prevalence ratio; LGB = lesbian, gay, and bisexual; LGBQ+ = lesbian, gay, bisexual, and queer

### Other

Other notable findings included prevalence estimates of 52.2% for alcohol use disorder and 41.3% for PTSD among sexual and gender minority (SGM) healthcare workers, while the lifetime prevalence of any mental health disorder among SGM sex workers was 72.3%. Alcohol abuse was more prevalent in lesbian compared to heterosexual physicians, while bisexual workers had a small increased risk of PTSD.

Other mental health outcomes were reported across seven studies (Table 5), three of which reported on alcohol and/or substance abuse disorders [28, 32, 33]. A significantly higher prevalence of alcohol use disorder was found among lesbian (prevalence of 8.6%) compared to heterosexual physicians (prevalence of 1.4%, *p* = 0.03) [28]. In a study of 102 sexual and gender minority (SGM) healthcare workers, although over half (52.2%) were found to have alcohol use disorder, this was not significantly higher than the 45.5% of non-SGM workers (aOR 1.355, 95% CI 0.839–2.189) [33]. Among frontline workers during the COVID-19 pandemic, none of the text conversations with 20 non-binary workers were flagged for substance abuse, resulting in an OR of 0 compared to binary workers [32]. Two studies reported on PTSD, one finding slightly higher PTSD symptoms in bisexual compared to monosexual workers [54], while another found no difference between SGM and non-SGM workers [33]. A study of 177 SGM sex workers found a prevalence of 72.3% for any self-reported mental health disorder [55], while a study of 105 lesbian, gay, bisexual, and transgender (LGBT) schoolteachers reported higher prevalence of seeking help for and time off work for depression or anxiety among village teachers compared to town or city teachers [56].

**Table 5:** Results of the included studies reporting on other mental health outcomes among LGBTQ+ workers

Author, year	Characteristics of LGBTQ+ workers	Outcomes	Methods for measuring outcomes	Pertinent findings
Brogan, 2003 [28]	115 lesbian physicians	Alcohol abuse/ dependence, eating disorders, substance abuse/ dependence	Self-report	Prevalence: Alcohol abuse/dependence 8.6% in lesbians, 1.4% in heterosexual. Eating disorders 7.9% in lesbians, 5.7% in heterosexual. Substance abuse/dependence 2.5% in lesbians, 0.8% in heterosexual. Comparison: Alcohol abuse/dependence *p* = 0.03, eating disorders *p* = 0.46, substance abuse/dependence *p* = 0.24.
Day, 2024 [54]	422 LGB workers aged 25 years and above	PTSD	17-item Post-Traumatic Stress Disorder Checklist	Comparison: Bisexuals higher in PTSD symptoms than monosexuals, although effect size small (*F* = 7.67, ρ = 0.00, observed power = 0.95, $\:{}_{\text{p}}^{2}$= 0.02).
Lee, 2019 [56]	105 LGBT schoolteachers	Seeking help for depression or anxiety, time off work for depression or anxiety	Self-report	Prevalence: Seeking help 61% of village teachers, 12.5% of town or city teachers. Time off work 46% of village teachers, 4% of town teachers, 7% of city teachers.
Puri, 2017 [55]	177 SGM sex workers	Any mental health disorder (depression, PTSD, anxiety, schizophrenia, borderline personality disorder, attention-deficit/ hyperactivity disorder, bipolar disorder, other diagnosis specified)	Self-report	Prevalence: 72.3%. Comparison: SGM vs. non-SGM aOR 2.56 (95% CI 1.72–3.81).
Sugg, 2021 [32]	20 non-binary and 61 transgender frontline or essential workers during COVID-19 pandemic who engaged with a text-based crisis service	Substance abuse	Daily text conversations flagged for substance abuse	Comparison: Non-binary vs. binary aOR 0.00 (95% CI 0.00–0.00), transgender vs. cisgender aOR 1.75 (95% CI 0.59–5.18).
Wojcik, 2022 [33]	102 SGM frontline healthcare workers	Alcohol use disorder, PTSD	PC-PTSD ≥ 3, AUDIT-C ≥ 3	Prevalence: PTSD 41.3% SGM, 33.5% non-SGM. Alcohol use disorder 52.2% SGM, 45.5% non-SGM. Comparison: PTSD aOR 1.355 (95% CI 0.839–2.189). Alcohol use disorder aOR 1.355 (95% CI 0.839–2.189).

aOR = adjusted odds ratio; AUDIT-C = Alcohol Use Disorders Identification Test-Concise; LGB = lesbian, gay, and bisexual; LGBT = lesbian, gay, bisexual, and transgender; PCL-S = PTSD Checklist– Specific version; PC-PTSD = Primary Care PTSD Screen for DSM-5; PTSD = post-traumatic stress disorder; SGM = sexual and gender minorities

### Risk of bias assessment

Of the included studies, four studies were rated at high risk of bias, six were assessed at moderate risk of bias, and the remaining 22 at low risk of bias (Supplementary Table 3). For the cross-sectional studies (*n* = 30), there was generally low bias across all categories, especially those relating to specification of inclusion criteria, description of subjects, measurement of exposure, and statistical analysis (Supplementary Fig. 1). Six studies were found to have high bias in measurement of outcomes, while five studies had high bias in management of confounding factors. For the cohort studies (*n* = 2), there was low risk of bias in all but three categories (Supplementary Fig. 2). Both cohort studies were rated at high risk of bias for participants being free of outcomes at the start of the study and there was high risk of bias for one of the studies in the categories of exposure measurement and statistical analysis.

## Discussion

Despite a comprehensive search of five databases, this review found limited evidence regarding the mental health of LGBTQ+ workers and the occupational groups studied were heterogeneous. Other than 13 studies in sex workers, we identified only 12 studies with research questions specific to LGBTQ+ workers’ mental health. Most studies reported higher prevalence of depression, anxiety, and suicidality or suicide attempts among LGBTQ+ compared to non-LGBTQ+ workers. Some associations of mental health disorders in select populations of LGBTQ+ workers included job stress, low income, heterosexism, low levels of supervisor support, and low social cohesion. No identified studies compared mental health between occupations, and no studies explored the effect of workplace interventions.

The findings of increased risk of depression, anxiety, and suicidality among LGBTQ+ compared to non-LGBTQ+ workers across several studies are consistent with the literature in the general LGBTQ+ population [9, 17, 57, 58]. LGBTQ+ community members are reported to be more likely to abuse alcohol and other substances [59]. However, one study of workers in our review found LGBTQ+ workers to be at higher risk of alcohol but not substance abuse [28], while two further studies found no association [32, 33]. These and other non-significant results may in part be due to the small sample sizes of LGBTQ+ workers in most of the included studies, making them underpowered to detect less-common outcomes. In the larger studies focused specifically on LGBTQ+ workers’ mental health, almost all mental health outcomes were more prevalent among LGBTQ+ workers with some large effect sizes observed [37, 48]. In contrast, when LGBTQ+ workers were not the primary cohort of interest, this left small numbers of self-identified LGBTQ+ individuals in general studies of workers. It is unknown if these studies facilitated the space required for workers to feel safe in disclosing their sexual or gender identity. Previous research indicates that many LGBTQ+ employees do not disclose their identity at work due to fear of discrimination and adverse workplace outcomes [60, 61]. As such, particularly in studies which did not specifically recruit LGBTQ+ workers, some LGBTQ+ workers may have been included in non-LGBTQ+ groups, weakening the mental health associations. The only study that specifically looked at workers questioning their sexual identity found that these individuals had some of the highest risks of suicidality [49]. This further reinforces this impact of misclassification as these individuals would have been classified within the non-LGBTQ+ category in studies where “questioning” was not an option.

As most studies were cross-sectional in nature, the causal relationships could not be investigated. One study including sex workers found that internalised stigma was significantly associated with depression and anxiety [27]. This was unsurprising given the well-established literature regarding LGBTQ+ employees’ experiences of stigma in the workplace [11, 62, 63]. Unlike social settings where LGBTQ+ individuals may have more opportunity to seek like-minded peers, workplace settings are largely outside the control of individual employees and leaving may not be financially feasible [64]. Heterosexism may also be pervasive in the workplace compared to other settings, given that most LGBTQ+ peoples’ work environments are likely to include some individuals who hold heterosexist attitudes [12], which may contribute to the link to depression among LGB workers [44]. The association between low supervisor support and depression in the study of gay and lesbian dual-earner couples [43] is consistent with studies linking supervisor support to greater wellbeing among working parents [65]. Other notable associations of adverse mental health outcomes in LGBTQ+ workers included job stress, which was also found to be an independent predictor of depression symptoms in a large prospective Belgian study [66], and low income, which has well-established links to depression [67].

We found more studies in sex workers, mostly transgender women, compared to all other specific occupations combined. It is estimated that between 24 and 75% of transgender women in the United States [68] and 54-80% in Asia [69] are involved in sex work. Transphobia limits access to sustainable employment opportunities for transgender women through job denial, demotion, workplace harassment and even workplace violence [70, 71]. Consequently, transgender women may be driven to sex work as a source of income [72].

Sex work itself can have profound impacts on mental health [73]. As such, it is likely that the nature of this work contributed equally if not more, to adverse psychological outcomes compared to sex workers’ sexual or gender identities, as suggested by the higher rates of depression we found in several studies of transgender or MSM sex workers compared to other transgender women or MSM. Another cohort studied was frontline workers, who face major stressors including excessive workload, sudden redeployment, fear of disease, and exposure to traumatic situations, making them vulnerable to mental health difficulties [74–76]. Studies of LGBTQ+ frontline workers have reported common experiences of stigma including disparaging remarks from colleagues, feelings of alienation, exclusion, and humiliation [77, 78]. Furthermore, unique stressors during the COVID-19 pandemic, such as being forced to live with non-affirming families and interruptions to affirming health or gender care, have been reported by LGBTQ+ communities, exacerbating poor mental health during these years [79, 80]. Farmers are another occupational group where LGBTQ+ individuals may be disproportionately affected by adverse mental health outcomes due to expectations of heteronormativity and exclusion in areas such as farm family succession planning [81, 82].

Although most findings were in line with the broader literature of LGBTQ+ mental health, some unexpected results should be acknowledged. Perhaps most notable was the negative association between sex work and suicidal ideation reported in Brazilian transgender women [53], discordant with all the other studies of sex workers. As the results were not adjusted for income, sex work may have served as a proxy for income in these transgender women who were otherwise excluded from the formal labour market, in part explaining the reduced likelihood of suicidality. Also, it has been previously hypothesised that, as it involves the recognition of and desire for transgender bodies, sex work may affirm transgender identity and serve as an element of social support, protecting against suicidal behaviour [83]. Another unexpected finding was that of higher levels of anxiety in gay compared to lesbian couples [43], given that women in the general population are more prone to anxiety disorders compared to men [84] and the same is true for lesbians compared to gay men [85]. The authors propose that gay fathers may experience higher levels of scrutiny as new parents, being both stereotyped as gay people to be less fit to parent than heterosexuals, plus stereotyped as men as being less nurturing than women [86].

The findings of this review should be considered alongside limitations of the included studies. It is not possible to make conclusions about the overall psychological wellbeing of LGBTQ+ workers from the results of these 32 studies given that the populations varied greatly. As we have described, the challenges for farmers are very different from those of sex workers or healthcare workers. Geographical variation in public opinion and legal frameworks, with same-sex activity still criminalised in some countries, may also exacerbate or mitigate the effects on LGBTQ+ workers’ mental health. Importantly, 30 of the 32 studies were cross-sectional in nature, meaning that directionality of association could not be explored. Quality assessment suggested that much of the available research was at high risk of bias. Another limitation was that over half of studies retrieved were in sex workers, which, although an important group for study, may not be particularly reflective of workers in other types of occupations, not least because sex work itself is associated with poor mental health.

The review itself also has some limitations. Gender identity is of course a complex issue, with ongoing debate regarding definitions and causal factors. For this review, we explored all terms currently in use as widely as possible, given that our aim was to review what was known about the mental health of LGBTQ+ people in the workplace. Our review is constrained by the definitions and methodologies adopted by the researchers in each study. Secondly, it was confined to studies in the English language, however, we still captured studies from 14 countries across Europe, Asia, North and South America, and Australia, representing a wide geographical sample and a diverse range of social acceptance and protections for LGBTQ+ people in the workplace [24]. The search was also limited to studies published since the year 2000. Results from earlier studies may have provided additional perspectives, however their relevance to today’s LGBTQ+ workforce might be limited given the vast shifts in public attitudes towards the LGBTQ+ community over the last two to three decades [87]. We also only included studies reporting on ICD-10 mental health outcomes. There is scope for future reviews to summarise the literature on additional mental health outcomes such as burnout and psychological distress, including data from qualitative studies. Finally, we were unable to perform meta-analyses given the heterogeneity of cohorts and methodologies. As further studies emerge regarding mental health of LGBTQ+ workers, the research question should be revisited.

One of the major gaps identified was the lack of studies comparing workers in different sectors. All studies either focused on a specific occupation (mostly sex workers), some broader industry areas (e.g., healthcare workers), or the general workforce. Future studies should involve LGBTQ+ workers across a range of industries and occupations to determine those at highest risk of mental health disorders and the workplace-specific factors implicated. As most studies were cross-sectional, there is a need for longitudinal studies to explore trends and whether shifting public sentiment, or different legal frameworks, translate to better health in LGBTQ+ workers. Additionally, the classification of sexual and gender minority groups should be considered. The umbrella term LGBTQ+ encompasses a diverse group of sexual and gender minorities with often different concerns and goals. Rather than combining these groups by default, future research should be co-designed with sexual and gender minority workers to ensure that the scope of, and questions in, research studies accurately reflect their identities and specific issues they face in the workplace. Importantly, no studies evaluated workplace interventions. Given the increasing movement towards programs such as diversity training aiming to improve LGBTQ+ worker wellbeing [88], determining the impact of these programs is paramount. Also, while some studies collected data about, and adjusted for, factors such as socioeconomic status and racial identity, no studies explored the impact of intersectionality. It is essential to understand how socioeconomic, ethnocultural, and other aspects of LGBTQ+ workers’ identities intersect in their experiences at work and mental health outcomes. Mental health professionals assessing LGBTQ+ patients are encouraged to explore their work experiences and choices with them, highlighting their workplace rights and encouraging and supporting them to obtain or maintain employment.

## Conclusions

The LGBTQ+ community is heterogeneous. This systematic review showed an increased risk of depression, anxiety, and suicidality in select populations of LGBTQ+ compared to non-LGBTQ+ workers. Other than studies of sex workers, whose psychological health is likely to be affected equally if not more by the nature of their work than their sexual or gender identity, few studies were conducted with the specific aim of understanding LGBTQ+ workers’ mental health. Almost all studies were cross-sectional in nature, and no studies compared industries or explored the effects of workplace interventions. Longitudinal research across industries is needed to understand the causes and burden of mental health disorders in LGBTQ+ workers, in order for effective solutions, including workplace interventions, to be developed and tested.

PROSPERO: CRD42024587605.

Clinical trial number: Not applicable.

## Electronic supplementary material

Below is the link to the electronic supplementary material.

Supplementary Material 1

## Data Availability

Data sharing is not applicable to this article as no datasets were generated or analysed during the current study.

## Abbreviations

GAD: Generalised anxiety disorder
GAD-7: Generalised anxiety disorder 7-item scale
HIV: Human immunodeficiency virus
ICD-10: International Classification of Diseases, Tenth Revision
LGB: Lesbian, gay, and bisexual
LGBQ+: Lesbian, gay, bisexual, and queer
LGBT: Lesbian, gay, bisexual, and transgender
LGBTQ+: Lesbian, gay, bisexual, transgender, queer, and other sexual and gender minorities
MSM: Men who have sex with men
OR: Odds ratio
PHQ: Patient Health Questionnaire
PR: Prevalence ratio
PRISMA: Preferred Reporting Items for Systematic Reviews and Meta-Analyses
PTSD: Post-traumatic stress disorder
RR: Relative risk
SGM: Sexual and gender minority
