# Supplementary material for: Mental health of LGBTQ+ workers: a systematic review
Source: BMC Psychiatry. 2025 Feb 11;25:114. doi: 10.1186/s12888-025-06556-2 (PMC11817621; doi:10.1186/s12888-025-06556-2)
Supplement: Supplementary file 1 — Supplementary Material 1 [file 12888_2025_6556_MOESM1_ESM.docx]

**Mental health of LGBTQ+ workers: A systematic review: Supplementary material**

**Supplementary Text 1: Search strategy of the systematic review.**

Database: Ovid MEDLINE® 1946 to Present with Daily Update

Search Strategy: 2000-2024 English language articles

1. exp “Sexual and Gender Minorities”/
2. exp Homosexuality/
3. Bisexuality/
4. Transsexualism/
5. “sexual orientation”.ti,ab
6. lgb*.ti,ab
7. gay.ti,ab
8. lesbian.ti,ab
9. bisexual*.ti,ab
10. transgender.ti,ab
11. 1 or 2 or 3 or 4 or 5 or 6 or 7 or 8 or 9 or 10
12. Workplace/
13. work*.ti,ab
14. occupation*.ti,ab
15. employee*.ti,ab
16. 12 or 13 or 14 or 15
17. Mental Health/
18. “mental health”.ti,ab
19. “psychological health”.ti,ab
20. exp Depressive Disorder/
21. depression.ti,ab
22. exp Anxiety Disorders/
23. anxiety.ti,ab
24. exp Substance-Related Disorders/
25. “alcohol abuse”.ti,ab
26. “alcohol us*”.ti,ab
27. “drug abuse”.ti,ab
28. “drug us*”.ti,ab
29. Suicide/
30. suicid*.ti,ab
31. Bipolar Disorder/
32. bipolar.ti,ab
33. Stress Disorders, Post-Traumatic/
34. ptsd.ti,ab
35. “post-traumatic stress disorder”.ti,ab
36. “posttraumatic stress disorder”.ti,ab
37. exp “Feeding and Eating Disorders”
38. anorexia.ti,ab
39. bulimia.ti,ab
40. exp Obsessive-Compulsive Disorder/
41. ocd.ti,ab
42. “obsessive-compulsive disorder”.ti,ab
43. exp Schizophrenia/
44. schizophrenia.ti,ab
45. exp Personality Disorders/
46. “personality disorder*”.ti,ab
47. 17 or 18 or 19 or 20 or 21 or 22 or 23 or 24 or 25 or 26 or 27 or 28 or 29 or 30 or 31 or 32 or 33 or 34 or 35 or 36 or 37 or 38 or 39 or 40 or 41 or 42 or 43 or 45
48. 11 and 16 and 47

Database: Embase Classic+Embase 1947 to Present with Daily Update

Search Strategy: 2000-2024 English language articles

1. exp “sexual and gender minority”/
2. exp homosexuality/
3. bisexuality/
4. transsexualism/
5. “sexual orientation”.ti,ab
6. lgb*.ti,ab
7. gay.ti,ab
8. lesbian.ti,ab
9. bisexual*.ti,ab
10. transgender.ti,ab
11. 1 or 2 or 3 or 4 or 5 or 6 or 7 or 8 or 9 or 10
12. workplace/
13. work*.ti,ab
14. occupation*.ti,ab
15. employee*.ti,ab
16. 12 or 13 or 14 or 15
17. mental health/
18. “mental health”.ti,ab
19. “psychological health”.ti,ab
20. exp depression/
21. depression.ti,ab
22. exp anxiety disorder/
23. anxiety.ti,ab
24. substance abuse/
25. “alcohol abuse”.ti,ab
26. “alcohol us*”.ti,ab
27. “drug abuse”.ti,ab
28. “drug us*”.ti,ab
29. suicide/
30. suicid*.ti,ab
31. exp bipolar disorder/
32. bipolar.ti,ab
33. posttraumatic stress disorder/
34. ptsd.ti,ab
35. “post-traumatic stress disorder”.ti,ab
36. “posttraumatic stress disorder”.ti,ab
37. exp eating disorder/
38. anorexia.ti,ab
39. bulimia.ti,ab
40. exp obsessive compulsive disorder/
41. ocd.ti,ab
42. “obsessive-compulsive disorder”.ti,ab
43. exp schizophrenia/
44. schizophrenia.ti,ab
45. exp personality disorder/
46. “personality disorder*”.ti,ab
47. 17 or 18 or 19 or 20 or 21 or 22 or 23 or 24 or 25 or 26 or 27 or 28 or 29 or 30 or 31 or 32 or 33 or 34 or 35 or 36 or 37 or 38 or 39 or 40 or 41 or 42 or 43 or 44 or 45 or 46
48. 11 and 16 and 47

Database: APA PsycInfo 1806 to Present with Daily Update

Search Strategy: 2000-2024 English language articles

1. exp Sexual Minority Groups/
2. exp LGBTQ/
3. “sexual orientation”.ti,ab
4. lgb*.ti,ab
5. gay.ti,ab
6. lesbian.ti,ab
7. bisexual*.ti,ab
8. transgender.ti,ab
9. 1 or 2 or 3 or 4 or 5 or 6 or 7 or 8
10. Occupations/
11. work*.ti,ab
12. occupation*.ti,ab
13. employee*.ti,ab
14. 10 or 11 or 12 or 13
15. Mental Health/
16. “mental health”.ti,ab
17. exp Major Depression/
18. depression.ti,ab
19. exp Anxiety Disorders/
20. anxiety.ti,ab
21. exp “Substance Use Disorder”/
22. “alcohol abuse”.ti,ab
23. “alcohol us*”.ti,ab
24. “drug abuse”.ti,ab
25. “drug us*”.ti,ab
26. Suicide/
27. suicid*.ti,ab
28. exp Bipolar Disorder/
29. bipolar.ti,ab
30. Posttraumatic Stress Disorder/
31. ptsd.ti,ab
32. “post-traumatic stress disorder”.ti,ab
33. “posttraumatic stress disorder”.ti,ab
34. exp Eating Disorders/
35. anorexia.ti,ab
36. bulimia.ti,ab
37. exp Obsessive Compulsive Disorder/
38. ocd.ti,ab
39. “obsessive-compulsive disorder”.ti,ab
40. exp Schizophrenia/
41. schizophrenia.ti,ab
42. exp Personality Disorders/
43. “personality disorder*”.ti,ab
44. 15 or 16 or 17 or 18 or 19 or 20 or 21 or 22 or 23 or 24 or 25 or 26 or 27 or 28 or 29 or 30 or 31 or 32 or 33 or 34 or 35 or 36 or 37 or 38 or 39 or 40 or 41 or 42 or 43
45. 9 and 14 and 44

Database: Scopus (Elsevier) 1788 to Present with Daily Update

Search Strategy: 2000-2024 English language articles

( ( ( TITLE ( "sexual orientation" ) OR ABS ( "sexual orientation" ) ) AND PUBYEAR > 1999 ) OR ( ( TITLE ( lgb* ) OR ABS ( lgb* ) ) AND PUBYEAR > 1999 ) OR ( ( TITLE ( gay ) OR ABS ( gay ) ) AND PUBYEAR > 1999 ) OR ( ( TITLE ( lesbian ) OR ABS ( lesbian ) ) AND PUBYEAR > 1999 ) OR ( ( TITLE ( bisexual* ) OR ABS ( bisexual* ) ) AND PUBYEAR > 1999 ) OR ( ( TITLE ( transgender ) OR ABS ( transgender ) ) AND PUBYEAR > 1999 ) ) AND ( ( ( TITLE ( work* ) OR ABS ( work* ) ) AND PUBYEAR > 1999 ) OR ( ( TITLE ( occupation* ) OR ABS ( occupation* ) ) AND PUBYEAR > 1999 ) OR ( ( TITLE ( employee* ) OR ABS ( employee* ) ) AND PUBYEAR > 1999 ) ) AND ( ( ( TITLE ( "mental health" ) OR ABS ( "mental health" ) ) AND PUBYEAR > 1999 ) OR ( ( TITLE ( "psychological health" ) OR ABS ( "psychological health" ) ) AND PUBYEAR > 1999 ) OR ( ( TITLE ( depression ) OR ABS ( depression ) ) AND PUBYEAR > 1999 ) OR ( ( TITLE ( anxiety ) OR ABS ( anxiety ) ) AND PUBYEAR > 1999 ) OR ( ( TITLE ( "alcohol abuse" ) OR ABS ( "alcohol abuse" ) ) AND PUBYEAR > 1999 ) OR ( ( TITLE ( "alcohol us*" ) OR ABS ( "alcohol us*" ) ) AND PUBYEAR > 1999 ) OR ( ( TITLE ( "drug abuse" ) OR ABS ( "drug abuse" ) ) AND PUBYEAR > 1999 ) OR ( ( TITLE ( "drug us*" ) OR ABS ( "drug us*" ) ) AND PUBYEAR > 1999 ) OR ( ( TITLE ( suicid* ) OR ABS ( suicid* ) ) AND PUBYEAR > 1999 ) OR ( ( TITLE ( bipolar ) OR ABS ( bipolar ) ) AND PUBYEAR > 1999 ) OR ( ( TITLE ( ptsd ) OR ABS ( ptsd ) ) AND PUBYEAR > 1999 ) OR ( ( TITLE ( "post-traumatic stress disorder" ) OR ABS ( "post-traumatic stress disorder" ) ) AND PUBYEAR > 1999 ) OR ( ( TITLE ( "posttraumatic stress disorder" ) OR ABS ( "posttraumatic stress disorder" ) ) AND PUBYEAR > 1999 ) OR ( ( TITLE ( "eating disorder*" ) OR ABS ( "eating disorder*" ) ) AND PUBYEAR > 1999 ) OR ( ( TITLE ( anorexia ) OR ABS ( anorexia ) ) AND PUBYEAR > 1999 ) OR ( ( TITLE ( bulimia ) OR ABS ( bulimia ) ) AND PUBYEAR > 1999 ) OR ( ( TITLE ( ocd ) OR ABS ( ocd ) ) AND PUBYEAR > 1999 ) OR ( ( TITLE ( "obsessive-compulsive disorder" ) OR ABS ( "obsessive-compulsive disorder" ) ) AND PUBYEAR > 1999 ) OR ( ( TITLE ( schizophrenia ) OR ABS ( schizophrenia ) ) AND PUBYEAR > 1999 ) OR ( ( TITLE ( "personality disorder*" ) OR ABS ( "personality disorder*" ) ) AND PUBYEAR > 1999 ) )

Database: CINAHL Complete 1962 to Present with Daily Update

Search Strategy: 2000-2024 English language articles

1. (MH “Sexual and Gender Minorities+”)
2. (MH "Homosexuality")
3. (MH "Bisexuality")
4. TI "sexual orientation" OR AB "sexual orientation"
5. TI gay OR AB gay
6. TI lgb* OR AB lgb*
7. TI lesbian OR AB lesbian
8. TI bisexual* OR AB bisexual*
9. TI transgender OR AB transgender
10. S1 OR S2 OR S3 OR S4 OR S5 OR S6 OR S7 OR S8 OR S9
11. (MH "Work Environment")
12. TI work* OR AB work*
13. TI occupation* OR AB occupation*
14. TI employee* OR AB employee*
15. S11 OR S12 OR S13 OR S14
16. (MH "Mental Health")
17. TI "mental health" OR AB "mental health"
18. TI "psychological health" OR AB "psychological health"
19. (MH "Depression+")
20. TI depression OR AB depression
21. (MH "Anxiety Disorders+")
22. TI anxiety OR AB anxiety
23. (MH "Substance Use Disorders+")
24. TI "alcohol abuse" OR AB "alcohol abuse"
25. TI "alcohol us*" OR AB "alcohol us*"
26. TI "drug abuse" OR AB "drug abuse"
27. TI "drug us*" OR AB "drug us*"
28. (MH "Suicide")
29. TI suicid* OR AB suicid*
30. (MH "Bipolar Disorder+")
31. TI bipolar OR AB bipolar
32. (MH "Stress Disorders, Post-Traumatic")
33. TI ptsd OR AB ptsd
34. TI "post-traumatic stress disorder" OR AB "post-traumatic stress disorder"
35. TI "posttraumatic stress disorder" OR AB "posttraumatic stress disorder"
36. (MH "Eating Disorders+")
37. TI anorexia OR AB anorexia
38. TI bulimia OR AB bulimia
39. (MH "Obsessive-Compulsive Disorder+")
40. TI ocd OR AB ocd
41. TI "obsessive-compulsive disorder" OR AB "obsessive-compulsive disorder"
42. (MH "Schizophrenia")
43. TI schizophrenia OR AB schizophrenia
44. (MH "Personality Disorders+")
45. TI "personality disorder*" OR AB "personality disorder*"
46. S16 OR S17 OR S18 OR S19 OR S20 OR S21 OR S22 OR S23 OR S24 OR S25 OR S26 OR S27 OR S28 OR S29 OR S30 OR S31 OR S32 OR S33 OR S34 OR S35 OR S36 OR S37 OR S38 OR S39 OR S40 OR S41 OR S42 OR S43 OR S44 OR S45
47. S10 AND S15 AND S46

**Supplementary Table 1: PRISMA checklist.**

| **Topic** | **Item #** | **Checklist item** | **Location where item is reported** |
| --- | --- | --- | --- |
| Title | 1 | Identify the report as a systematic review. | Page 1 |
| Abstract | 2 | See the PRISMA 2020 for Abstracts checklist. | Page 1 |
| Rationale | 3 | Describe the rationale for the review in the context of existing knowledge. | Page 3 |
| Objectives | 4 | Provide an explicit statement of the objective(s) or question(s) the review addresses. | Page 4 |
| Eligibility criteria | 5 | Specify the inclusion and exclusion criteria for the review and how studies were grouped for the syntheses. | Page 5 |
| Information sources | 6 | Specify all databases, registers, websites, organisations, reference lists and other sources searched or consulted to identify studies. Specify the date when each source was last searched or consulted. | Page 4 |
| Search strategy | 7 | Present the full search strategies for all databases, registers and websites, including any filters and limits used. | Page 4, Appendix page 1 |
| Selection process | 8 | Specify the methods used to decide whether a study met the inclusion criteria of the review, including how many reviewers screened each record and each report retrieved, whether they worked independently, and if applicable, details of automation tools used in the process. | Page 6 |
| Data collection process | 9 | Specify the methods used to collect data from reports, including how many reviewers collected data from each report, whether they worked independently, any processes for obtaining or confirming data from study investigators, and if applicable, details of automation tools used in the process. | Page 6 |
| Data items | 10 | List and define all outcomes for which data were sought. Specify whether all results that were compatible with each outcome domain in each study were sought (e.g. for all measures, time points, analyses), and if not, the methods used to decide which results to collect. | Page 7 |
| Study risk of bias assessment | 11 | Specify the methods used to assess risk of bias in the included studies, including details of the tool(s) used, how many reviewers assessed each study and whether they worked independently, and if applicable, details of automation tools used in the process. | Page 7 |
| Effect measures | 12 | Specify for each outcome the effect measure(s) (e.g. risk ratio, mean difference) used in the synthesis or presentation of results. | Page 7 |
| Synthesis methods | 13 | Describe the processes used to decide which studies were eligible for each synthesis (e.g. tabulating the study intervention characteristics and comparing against the planned groups for each synthesis (item #5)). | Page 6 |
| Reporting bias assessment | 14 | Describe any methods used to assess risk of bias due to missing results in a synthesis (arising from reporting biases). | Page 7 |
| Certainty assessment | 15 | Describe any methods used to assess certainty (or confidence) in the body of evidence for an outcome. | Page 7 |
| Study selection | 16 | Describe the results of the search and selection process, from the number of records identified in the search to the number of studies included in the review, ideally using a flow diagram. | Page 7, Figure 1 |
| Study characteristics | 17 | Cite each included study and present its characteristics. | Table 1 |
| Risk of bias in studies | 18 | Present assessments of risk of bias for each included study. | Page 13, Appendix Table 2, Appendix Figures 1-2 |
| Results of individual studies | 19 | For all outcomes, present, for each study: (a) summary statistics for each group (where appropriate) and (b) an effect estimate and its precision (e.g. confidence/credible interval), ideally using structured tables or plots. | Tables 2-5 |
| Results of syntheses | 20 | For each synthesis, briefly summarise the characteristics and risk of bias among contributing studies. | Page 13, Table 1 |
| Reporting biases | 21 | Present assessments of risk of bias due to missing results (arising from reporting biases) for each synthesis assessed. | Page 13, Appendix Figures 1-2 |
| Certainty of evidence | 22 | Present assessments of certainty (or confidence) in the body of evidence for each outcome assessed. | Tables 2-5 |
| Discussion | 23a | Provide a general interpretation of the results in the context of other evidence. | Page 15 |
|  | 23b | Discuss any limitations of the evidence included in the review. | Page 19 |
|  | 23c | Discuss any limitations of the review processes used. | Page 20 |
|  | 23d | Discuss implications of the results for practice, policy, and future research. | Page 21 |
| Registration and protocol | 24 | Provide registration information for the review, including register name and registration number, or state that the review was not registered. | Page 6 |
| Support | 25 | Describe sources of financial or non-financial support for the review, and the role of the funders or sponsors in the review. | Page 24 |
| Competing interests | 26 | Declare any competing interests of review authors. | Page 24 |
| Availability of data, code and other materials | 27 | Report which of the following are publicly available and where they can be found: template data collection forms; data extracted from included studies; data used for all analyses; analytic code; any other materials used in the review. | Page 24 |

**Supplementary Table 2: List of the 51 articles excluded at the full-text level by reason of exclusion.**

| **Reason for exclusion** | **Excluded articles** |
| --- | --- |
| Wrong study design (n=5) | Trudel-Fitzgerald 2016 (1), Cancela 2024 (2), Wypler 2020 (3), Reece-Nguyen 2022 (4), Oliveira 2024 (5) |
| No LGBTQ+-specific results (n=2) | Rosander 2023 (6), Maclin 2023 (7) |
| No worker-specific results (n=23) | Fisher 2023 (8), Dumas 2023 (9), Randall 2017 (10), Keuroghlian 2015 (11), Munoz-Laboy 2015 (12), Guzman-Parra 2016 (13), Wang 2014 (14), Li 2022 (15), Sartaj 2021 (16), Watson 2022 (17), Pagliaccio 2024 (18), Cramer 2022 (19), Drabble 2022 (20), Witte 2020 (21), Robles 2024 (22), Pandey 2022 (23), Doshi 2020 (24), Gates 2020 (25), Nemoto 2011 (26), Liu 2018 (27), Uyar 2023 (28), Zegarra-López 2023 (29), Wang 2020 (30) |
| Outcomes not mental health disorders (n=16) | Drydakis 2017 (31), Nawyn 2000 (32), Lloren 2017 (33), Barbee 2023 (34), Paterson 2023 (35), Senreich 2020 (36), She 2021 (37), Kim 2019 (38), Cox 2023 (39), Owens 2022 (40), Nowack 2020 (41), Boitet 2023 (42), Wang 2022 (43), Douglas 2022 (44), Dhanani 2024 (45), Landes 2023 (46) |
| LGBTQ+ and worker analyses not combined (n=5) | Gouse 2023 (47), Paredes Rivera 2024 (48), Trang 2024 (49), Legleye 2010 (50), Drydakis 2022 (51) |

**Supplementary Table 3: Overall risk of bias assessment for the 33 included studies.**

| **Author, year** | **Risk of bias** |
| --- | --- |
| Ali, 2023 (52) | Low |
| Amsalem, 2023 (53) | High |
| Bar-Johnson, 2014 (54) | Moderate |
| Brogan, 2003 (55) | Moderate |
| Chandler, 2021 (56) | Low |
| Chang, 2019 (57) | Low |
| Cuthbertson, 2024 (58) | Moderate |
| Day, 2024 (59) | Low |
| de Mattos Russo Rafael, 2021 (60) | Low |
| Goldberg, 2013 (61) | Low |
| Goldenberg, 2021 (62) | Low |
| Griffin, 2023 (63) | Low |
| Klare, 2021 (64) | Low |
| Kyron, 2021 (65) | Low |
| Lee, 2019 (66) | High |
| Logie, 2017 (67) | Moderate |
| Luz, 2024 (68) | Low |
| Moya, 2020 (69) | Low |
| Nuttbrock, 2014 (70) | Low |
| Puri, 2017 (71) | Moderate |
| Rashid, 2023 (72) | High |
| Renkiewicz, 2022 (73) | Low |
| Scoresby, 2023 (74) | Low |
| She, 2022 (75) | High |
| Smith, 2004 (76) | Low |
| Srivastava, 2022 & 2023 (77, 78) | Low |
| Sugg, 2021 (79) | Moderate |
| Teoh, 2023 (80) | Low |
| Thirunavukkarasu, 2021 (81) | Low |
| Wojcik, 2022 (82) | Low |
| Yan, 2014 (83) | Low |
| Yasami, 2023 (84) | Low |

**Supplementary Figure 1: Detailed risk of bias results of cross-sectional studies.**

**
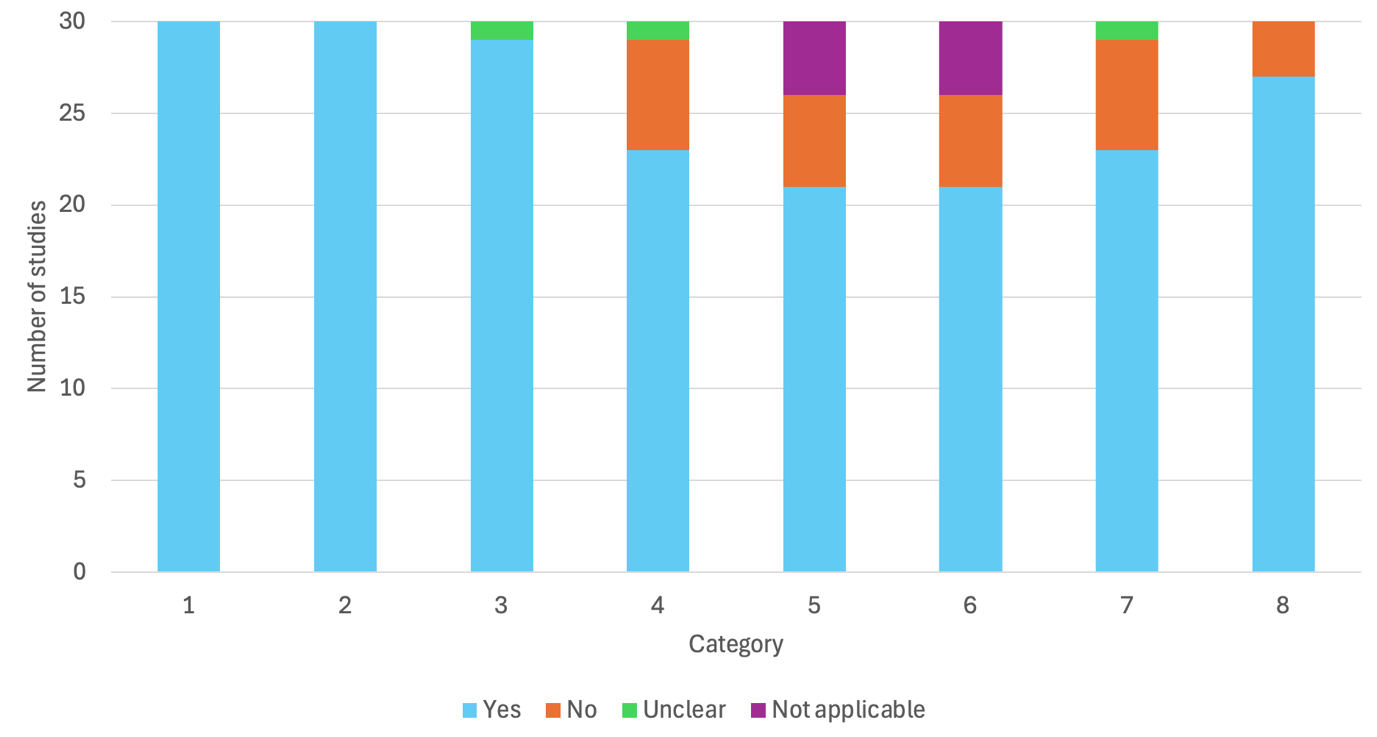
**

Categories:

1. Were the criteria for inclusion in the sample clearly defined?
2. Were the study subjects and the setting described in detail?
3. Was the exposure measured in a valid and reliable way?
4. Were objective, standard criteria used for measurement of the condition?
5. Were confounding factors identified?
6. Were strategies to deal with confounding factors stated?
7. Were the outcomes measured in a valid and reliable way?
8. Was appropriate statistical analysis used?

**Supplementary Figure 2: Detailed risk of bias results of cohort studies.**


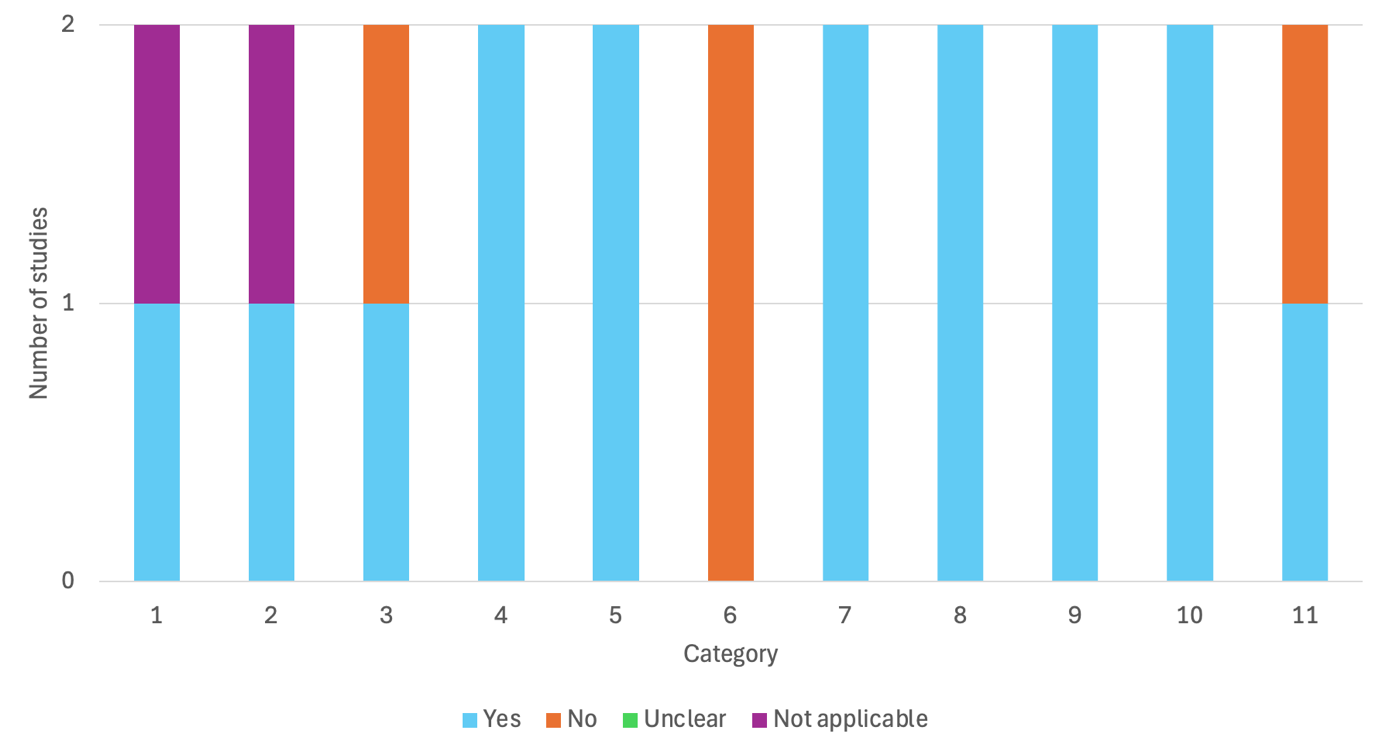


Categories:

1. Were the two groups similar and recruited from the same population?
2. Were the exposures measured similarly to assign people to both exposed and unexposed groups?
3. Was the exposure measured in a valid and reliable way?
4. Were confounding factors identified?
5. Were strategies to deal with confounding factors stated?
6. Were the groups/participants free of the outcome at the start of the study (or at the moment of exposure)?
7. Were the outcomes measured in a valid and reliable way?
8. Was the follow up time reported and sufficient to be long enough for outcomes to occur?
9. Was follow up complete, and if not, were the reasons to loss to follow up described and explored?
10. Were strategies to address incomplete follow up utilized?
11. Was appropriate statistical analysis used?

1. Trudel-Fitzgerald C, Chen Y, Singh A, Okereke OI, Kubzansky LD. Psychiatric, Psychological, and Social Determinants of Health in the Nurses' Health Study Cohorts. American journal of public health. 2016;106(9):1644-9.

2. Cancela D, Stutterheim SE, Uitdewilligen S. The Workplace Experiences of Transgender and Gender Diverse Employees: A Systematic Literature Review Using the Minority Stress Model. Journal of homosexuality. 2024:1-29.

3. Wypler J, Hoffelmeyer M. LGBTQ+ Farmer Health in COVID-19. Journal of agromedicine. 2020;25(4):370-3.

4. Reece-Nguyen T, Afonso AM, Vinson AE. Burnout, Mental Health, and Workplace Discrimination in Lesbian, Gay, Bisexual, Transgender, Queer/Questioning, Intersex, and Asexual Anesthesiologists. Anesthesiology clinics. 2022;40(2):245-55.

5. Oliveira A, Pereira H, Alckmin-Carvalho F. Occupational Health, Psychosocial Risks and Prevention Factors in Lesbian, Gay, Bisexual, Trans, Queer, Intersex, Asexual, and Other Populations: A Narrative Review. Societies. 2024;14(8).

6. Rosander M, Hetland J, Einarsen SV. Workplace bullying and mental health problems in balanced and gender-dominated workplaces. Work & Stress. 2023;37(3):325-44.

7. Maclin BJ, Wang Y, Rodriguez-Diaz C, Donastorg Y, Perez M, Gomez H, et al. Comparing typologies of violence exposure and associations with syndemic health outcomes among cisgender and transgender female sex workers living with HIV in the Dominican Republic. PloS one. 2023;18(9):e0291314.

8. Fisher MR, Turner C, McFarland W, Breslow AS, Wilson EC, Arayasirikul S. Through a Different Lens: Occupational Health of Sex-Working Young Trans Women. Transgender Health. 2023;8(2):200-6.

9. Dumas RE, Pepper CM. Bisexual-specific minority stress in nonsuicidal self-injury: The mediating role of perceived burdensomeness. Journal of Affective Disorders Reports. 2023;13:100608.

10. Randall AK, Totenhagen CJ, Walsh KJ, Adams C, Tao C. Coping with workplace minority stress: Associations between dyadic coping and anxiety among women in same-sex relationships. Journal of lesbian studies. 2017;21(1):70-87.

11. Keuroghlian AS, Reisner SL, White JM, Weiss RD. Substance use and treatment of substance use disorders in a community sample of transgender adults. Drug and alcohol dependence. 2015;152:139-46.

12. Munoz-Laboy M, Ripkin A, Garcia J, Severson N. Family and Work Influences on Stress, Anxiety and Depression Among Bisexual Latino Men in the New York City Metropolitan Area. Journal of immigrant and minority health. 2015;17(6):1615-26.

13. Guzman-Parra J, Sanchez-Alvarez N, de Diego-Otero Y, Perez-Costillas L, Esteva de Antonio I, Navais-Barranco M, et al. Sociodemographic Characteristics and Psychological Adjustment Among Transsexuals in Spain. Archives of sexual behavior. 2016;45(3):587-96.

14. Wang J, Hausermann M, Weiss MG. Mental health literacy and the experience of depression in a community sample of gay men. Journal of affective disorders. 2014;155:200-7.

15. Li W, Wang Y, Xu M, Liao Y, Zhou H, Ma H, et al. Temporal Trends and Differences in Sexuality among Depressed and Non-Depressed Adults in the United States. International journal of environmental research and public health. 2022;19(21).

16. Sartaj D, Krishnan V, Rao R, Ambekar A, Dhingra N, Sharan P. Mental illnesses and related vulnerabilities in the Hijra community: A cross-sectional study from India. The International journal of social psychiatry. 2021;67(3):290-7.

17. Watson C, Tatnell R. Resilience and non-suicidal self-injury in LGBTQIA+ people: Targets for prevention and intervention. Current Psychology: A Journal for Diverse Perspectives on Diverse Psychological Issues. 2022;41(1):307-14.

18. Pagliaccio D. Workplace experiences of LGBTQIA+ trainees, staff, and faculty in academic psychology, psychiatry, and neuroscience departments. Psychology and Sexuality. 2024.

19. Cramer RJ, Kaniuka AR, Yada FN, Diaz-Garelli F, Hill RM, Bowling J, et al. An analysis of suicidal thoughts and behaviors among transgender and gender diverse adults. Social psychiatry and psychiatric epidemiology. 2022;57(1):195-205.

20. Drabble LA, Mericle AA, Munroe C, Wootton AR, Trocki KF, Hughes TL. Examining Perceived Effects of Same-Sex Marriage Legalization Among Sexual Minority Women: Identifying Demographic Differences and Factors Related to Alcohol Use Disorder, Depression, and Self-Perceived Health. Sexuality Research and Social Policy. 2022;19(3):1285-99.

21. Witte TK, Kramper S, Carmichael KP, Chaddock M, Gorczyca K. A survey of negative mental health outcomes, workplace and school climate, and identity disclosure for lesbian, gay, bisexual, transgender, queer, questioning, and asexual veterinary professionals and students in the United States and United Kingdom. Journal of the American Veterinary Medical Association. 2020;257(4):417-31.

22. Robles G, Lee YG, Hillesheim J, Brusche D, Lopez-Matos J, Cain D, et al. Peer social support moderates the impact of ethnoracial discrimination on mental health among young sexual minority men of color. Journal of Social and Personal Relationships. 2024;41(9):2455-77.

23. Pandey AK, Seelman K. History of Sex Work Is Associated with Increased Risk of Adverse Mental Health and Substance Use Outcomes in Transgender Adults. International journal of environmental research and public health. 2022;19(23).

24. Doshi M, Macharia P, Mathenge J, Musyoki H, Amico KR, Battacharjee P, et al. Beyond biomedical and comorbidity approaches: Exploring associations between affinity group membership, health and health seeking behaviour among MSM/MSW in Nairobi, Kenya. Global public health. 2020;15(7):968-84.

25. Gates TG, Dentato MP. Volunteerism, mental health, and well-being in the lesbian, gay and bisexual community. Social Work in Mental Health. 2020;18(6):670-83.

26. Nemoto T, BÃ¶deker B, Iwamoto M. Social Support, Exposure to Violence and Transphobia, and Correlates of Depression Among Male-to-Female Transgender Women With a History of Sex Work. American Journal of Public Health. 2011;101(10):1980-8.

27. Liu Y, Jiang C, Li S, Gu Y, Zhou Y, An X, et al. Association of recent gay-related stressful events with depressive symptoms in Chinese men who have sex with men. BMC psychiatry. 2018;18(1):217.

28. Uyar B, Yucel I, Uyar E, Ates Budak E, Kelle I, Bulbuloglu S. A case-control study on depression, anxiety, and belief in sexual myths in trans women. Frontiers in Psychiatry. 2023;13:955577.

29. Zegarra-López AC, Garcia-Rabines D, Okumura-Clark A. Factors associated with depression in Peruvian LGBT + individuals exposed to discrimination and violence. Journal of Gay and Lesbian Social Services. 2023;35(4):511-37.

30. Wang Q, Chang R, Wang Y, Jiang X, Zhang S, Shen Q, et al. Correlates of alcohol and illicit drug use before commercial sex among transgender women with a history of sex work in China. Sexual health. 2020;17(1):45-52.

31. Drydakis N. Trans employees, transitioning, and job satisfaction. Journal of Vocational Behavior. 2017;98:1-16.

32. Nawyn SJ, Richman JA, Rospenda KM, Hughes TL. Sexual identity and alcohol-related outcomes: contributions of workplace harassment. Journal of substance abuse. 2000;11(3):289-304.

33. Lloren A, Parini L. How LGBT-supportive workplace policies shape the experience of lesbian, gay men, and bisexual employees. Sexuality Research & Social Policy: A Journal of the NSRC. 2017;14(3):289-99.

34. Barbee H, McKay T. Do supportive work environments matter for minority aging? Work stress and subjective cognitive impairment among middle-age and older lesbian, gay, bisexual, transgender, and queer adults. Acta psychologica. 2023;237:103949.

35. Paterson E, Paterson NAB, Ferris LJ. Mental health and well-being of anaesthetists during the COVID-19 pandemic: a scoping review. Anaesthesia. 2023;78(2):197-206.

36. Senreich E, Straussner SLA, Cooper CE. Health, wellness, and workplace experiences of lesbian, gay, and bisexual social workers. Journal of Gay & Lesbian Social Services: The Quarterly Journal of Community & Clinical Practice. 2020;32(2):209-39.

37. She R, Mo PKH, Ma T, Liu Y, Lau JTF. Impact of Minority Stress and Poor Mental Health on Sexual Risk Behaviors among Transgender Women Sex Workers in Shenyang, China. AIDS and behavior. 2021;25(6):1790-9.

38. Kim SY, Velez B, Daheim J, Lei N. Validation of the Work Family Conflict Scale for sexual minority employees. Journal of Career Assessment. 2019;27(4):594-609.

39. Cox EA, Burchell D, Bonnell K, Gauthier CA, Smilovsky K, Meunier S, et al. The Impact of Multiple Marginalized Social Statuses: How Being a Sexual Minority, a Woman, or Living with Low Income Relates to Workers' Well-being. Canadian Journal of Administrative Sciences. 2023;40(3):309-25.

40. Owens B, Mills S, Lewis N, Guta A. Work-related stressors and mental health among LGBTQ workers: Results from a cross-sectional survey. PloS one. 2022;17(10):e0275771.

41. Nowack V, Donahue JJ. Outcomes associated with employee and organisational LGBT value discrepancies. Psychology & Sexuality. 2020;11(1-2):32-44.

42. Boitet LM, Meese KA, Hays MM, Gorman CA, Sweeney KL, Rogers DA. Burnout, Moral Distress, and Compassion Fatigue as Correlates of Posttraumatic Stress Symptoms in Clinical and Nonclinical Healthcare Workers. Journal of Healthcare Management. 2023;68(6):427-51.

43. Wang J, Wicks D, Zhang C. Job-related well-being of sexual minorities: Evidence from the British workplace employment relations study. British Journal of Industrial Relations. 2022;60(4):841-63.

44. Douglas HM, Settles IH, Cech EA, Montgomery GM, Nadolsky LR, Hawkins AK, et al. Disproportionate impacts of COVID-19 on marginalized and minoritized early-career academic scientists. PLoS ONE. 2022;17(9 September):e0274278.

45. Dhanani LY, Totton R, Hall TK. Every action has a reaction: A model of coworker reactions to sexual minority employees' identity disclosure. Journal of Occupational & Organizational Psychology. 2024;97(2):602-22.

46. Landes SJ, Jaffe AE, McBain SA, Feinstein BA, Rhew IC, Kaysen DL. Prospective predictors of work limitations in young adult lesbian and bisexual women: An examination of minority stress, trauma exposure, and mental health. Stigma and Health. 2023;8(2):232-42.

47. Gouse BM, Schwarz AG, Gibbs JS, Weinberg JM, Yue H, Chava A, et al. Demographic predictors of lack of current mental health treatment among university students with a schizophrenia spectrum disorder. Early intervention in psychiatry. 2023;17(12):1207-15.

48. Paredes Rivera A, Drame AS, Knight LD. A 3-Year Retrospective Review of Complex Suicides With a Review of the Literature. The American journal of forensic medicine and pathology. 2024.

49. Trang D, Swafford CE, Kreps TA, Vance SD, Davidson J, Filiberto J, et al. A survey of the severity of mental health symptoms in the planetary science community. Nature Astronomy. 2024;8(6):691-6.

50. Legleye S, Beck F, Peretti-Watel P, Chau N, Firdion JM. Suicidal ideation among young French adults: association with occupation, family, sexual activity, personal background and drug use. Journal of affective disorders. 2010;123(1-3):108-15.

51. Drydakis N. Social Rejection, Family Acceptance, Economic Recession, and Physical and Mental Health of Sexual Minorities. Sexuality Research and Social Policy. 2022;19(3):1318-40.

52. Ali U, Ali SA. Prevalence of Depression and Subjective Job Stress Among Men who have Sex with Men and Transgender Community Health Workers in Pakistan. J Coll Physicians Surg Pak. 2023;33(7):779-83.

53. Amsalem D, Fisch CT, Wall M, Choi CJ, Lazarov A, Markowitz JC, et al. Anxiety and Depression Symptoms Among Young U.S. Essential Workers During the COVID-19 Pandemic. Psychiatr Serv. 2023;74(10):1010-8.

54. Bar-Johnson M, Weiss P. Mental health and sexual identity in a sample of male sex workers in the Czech Republic. Med Sci Monit. 2014;20:1682-6.

55. Brogan DJ, O'Hanlan KA, Elon L, Frank E. Health and professional characteristics of lesbian and heterosexual women physicians. J Am Med Womens Assoc (1972). 2003;58(1):10-9.

56. Chandler CJ, Meunier É, Eaton LA, Andrade E, Bukowski LA, Matthews DD, et al. Syndemic Health Disparities and Sexually Transmitted Infection Burden Among Black Men Who Have Sex with Men Engaged in Sex Work in the U.S. Arch Sex Behav. 2021;50(4):1627-40.

57. Chang R, Wang H, She R, Zhang S, Tsamlag L, Shen Q, et al. Feelings of Entrapment and Defeat Mediate the Association Between Self-Esteem and Depression Among Transgender Women Sex Workers in China. Front Psychol. 2019;10:2241.

58. Cuthbertson C, Rivas-Koehl D, Codamon A, Billington A, Rivas-Koehl M. Mental Health Among LGBTQ+ Farmers in the United States. J Agromedicine. 2024;29(4):583-93.

59. Day NE, Meglich P, Porter TH. Comparing the relationship of workplace bullying and PTSD in bisexual versus monosexual workers. Psychology of Sexual Orientation and Gender Diversity. 2024;11(1):126-38.

60. Rafael RMR, Jalil EM, Luz PM, de Castro CRV, Wilson EC, Monteiro L, et al. Prevalence and factors associated with suicidal behavior among trans women in Rio de Janeiro, Brazil. PLoS One. 2021;16(10):e0259074.

61. Goldberg AE, Smith JZ. Work Conditions and Mental Health in Lesbian and Gay Dual-Earner Parents. Family Relations. 2013;62(5):727-40.

62. Goldenberg T, Kerrigan D, Gomez H, Perez M, Donastorg Y, Barrington C. Stigma, Social Cohesion, and Mental Health Among Transgender Women Sex Workers Living with HIV in the Dominican Republic. Stigma Health. 2021;6(4):467-75.

63. Griffin M, Jaiswal J, Olsson T, Gui J, Stults CB, Halkitis PN. Depression Severity among a Sample of LGBTQ+ Individuals during the COVID-19 Pandemic. Societies [Internet]. 2023; 13(11).

64. Klare D, Finch A, Arreola A, Dailey S, Howard K. Examining how sexual identity, psychosocial factors, and organizational differences relate to intent-to-quit in a large-scale, cross-sectional study. Journal of Gay & Lesbian Social Services. 2021;33(4):493-511.

65. Kyron MJ, McEvoy PM, Gilbey D, Lin A, Mazza C, Rikkers W, et al. Sexual orientation and prevalence of mental health difficulties among emergency services employees. J Affect Disord. 2021;287:240-6.

66. Lee C. How do Lesbian, Gay and Bisexual Teachers Experience UK Rural School Communities? Social Sciences [Internet]. 2019; 8(9).

67. Logie CH, Wang Y, Lacombe-Duncan A, Jones N, Ahmed U, Levermore K, et al. Factors associated with sex work involvement among transgender women in Jamaica: a cross-sectional study. J Int AIDS Soc. 2017;20(1):21422.

68. Luz LS, Cassenote AJF, Valente EP, Mariani I, Lazzerini M, Lima C, et al. Brazilian Physicians Mental Health: A Cross-Sectional Nationwide Study exploring factors associated with prevalence of suicide planning and attempts. Braz J Psychiatry. 2024;46.

69. Discrimination, work stress, and psychological well-being in LGBTI workers in Spain [press release]. Spain: Colegio Oficial de Psicólogos de Madrid2020.

70. Nuttbrock L, Bockting W, Rosenblum A, Hwahng S, Mason M, Macri M, et al. Gender abuse, depressive symptoms, and substance use among transgender women: a 3-year prospective study. Am J Public Health. 2014;104(11):2199-206.

71. Puri N, Shannon K, Nguyen P, Goldenberg SM. Burden and correlates of mental health diagnoses among sex workers in an urban setting. BMC Womens Health. 2017;17(1):133.

72. Rashid A, Afiqah SN. Depression, Anxiety, and Stress among the Malay Muslim Transgender Women in Northern Malaysia: A Mixed-Methods Study. Issues Ment Health Nurs. 2023;44(11):1124-32.

73. Renkiewicz GK, Hubble MW. Secondary Traumatic Stress in Emergency Services Systems Project: Quantifying the Effect of Personal Trauma Profiles on Lifetime Prevalence of Suicidality in Emergency Medical Services Personnel. Air Med J. 2022;41(5):463-72.

74. Scoresby K, Jurney C, Fackler A, Tran CV, Nugent W, Strand E. Relationships between diversity demographics, psychological distress, and suicidal thinking in the veterinary profession: a nationwide cross-sectional study during COVID-19. Front Vet Sci. 2023;10:1130826.

75. She R, Mo PKH, Cai Y, Ma T, Liu Y, Lau JTF. Mental health service utilisation among transgender women sex workers who are at risk of mental health problems in Shenyang, China: An application of minority stress theory. Health Soc Care Community. 2022;30(4):e981-e93.

76. Smith NG, Ingram KM. Workplace Heterosexism and Adjustment Among Lesbian, Gay, and Bisexual Individuals: The Role of Unsupportive Social Interactions. American Psychological Association; 2004. p. 57-67.

77. Srivastava A, Davis JP, Patel P, Daniel EE, Karkal S, Rice E. Polyvictimization, Sex Work, and Depressive Symptoms Among Transgender Women and Men Who Have Sex With Men. J Interpers Violence. 2022;37(13-14):Np11089-np109.

78. Srivastava A, Davis JP, Patel P, Daniel EE, Karkal S, Rice E. Sex work, gender transition, family rejection and depressive symptoms among transgender women in India. Int J Transgend Health. 2023;24(1):49-58.

79. Sugg MM, Runkle JD, Andersen L, Weiser J, Michael KD. Crisis response among essential workers and their children during the COVID-19 pandemic. Prev Med. 2021;153:106852.

80. Teoh KR, Dunning A, Taylor AK, Gopfert A, Chew-Graham CA, Spiers J, et al. Working conditions, psychological distress and suicidal ideation: cross-sectional survey study of UK junior doctors. BJPsych Open. 2023;10(1):e14.

81. Thirunavukkarasu B, Khandekar J, Parasha M, Dhiman B, Yadav K. Psychosocial health and its associated factors among Men who have sex with Men in India: A cross-sectional study. Indian J Psychiatry. 2021;63(5):490-4.

82. Wojcik H, Breslow AS, Fisher MR, Rodgers CRR, Kubiszewski P, Gabbay V. Mental Health Disparities Among Sexual and Gender Minority Frontline Health Care Workers During the Height of the COVID-19 Pandemic. LGBT Health. 2022;9(5):359-67.

83. Yan H, Wong FY, Zheng T, Ning Z, Ding Y, Nehl EJ, et al. Social support and depressive symptoms among 'money' boys and general men who have sex with men in Shanghai, China. Sex Health. 2014;11(3):285-7.

84. Yasami M, Zhu H, Dewan M. Poverty, Psychological Distress, and Suicidality Among Gay Men and Transgender Women Sex Workers During the Covid-19 Pandemic in Phuket, Thailand. Sex Res Social Policy. 2023:1-17.
